# Structural and functional characterization of the brain-specific dynamin superfamily member RNF112

**DOI:** 10.1073/pnas.2419449122

**Published:** 2025-04-08

**Authors:** Ya-Ting Zhong, Li-Li Huang, Kangning Li, Bingke Yang, Xueting Ye, Hao-Ran Zhong, Bing Yu, Menghan Ma, Yuerong Yuan, Yang Meng, Runfeng Pan, Haiqing Zhang, Lijun Shi, Yunyun Wang, Ruijun Tian, Song Gao, Xin Bian

**Affiliations:** ^a^Guangdong Provincial Clinical Research Center for Cancer, State Key Laboratory of Oncology in South China, Sun Yat-sen University Cancer Center, Guangzhou, Guangdong 510060, China; ^b^State Key Laboratory of Medicinal Chemical Biology, College of Life Sciences, Frontiers Science Center for Cell Responses, Nankai University, Tianjin 300071, China; ^c^Department of Chemistry and Research Center for Chemical Biology and Omics Analysis, College of Science, Southern University of Science and Technology, Shenzhen 518055, China; ^d^College of Life Sciences, Nankai University, Tianjin 300071, China; ^e^Integrated Traditional Chinese and Western Medicine Research Center, Sun Yat-sen University Cancer Center, Guangzhou, Guangzhou, Guangdong 510060, China; ^f^National Laboratory of Biomacromolecules, Institute of Biophysics, Chinese Academy of Sciences, Beijing 100101, China

**Keywords:** dynamin superfamily, membrane remodeling, protein structure

## Abstract

Most members of the dynamin superfamily of large guanosine triphophatases (GTPases) have an ability to remodel membranes in response to guanosine triphosphate (GTP) hydrolysis. Ring Finger Protein 112 (RNF112) (ZNF179/neurolastin) is a recently identified brain-specific dynamin-like protein possessing a really interesting new gene (RING) finger domain. Despite its essential role as an E3 ligase in neuron development, the architecture of RNF112 and the exact role of its GTPase activity remain unknown. Here, we determined the crystal structure of truncated RNF112 (RNF112_T_) containing a GTPase domain (GD) and three-helical middle domain (MD) at different nucleotide-loading states. In the nucleotide-free (apo) state, the monomeric RNF112_T_ remained in a unique self-restraint conformation characterized by docking of the proximal end of the MD to a groove in the GD. At the transition state of GTP hydrolysis, the MD was released from the GD and stretched aside to form an intertwined RNF112_T_ homodimer. Engineered RNF112 equipped with the C-terminal elements of ATL1 or the two transmembrane domains of yeast Sac1p relocated to the endoplasmic reticulum and was capable of mediating membrane remodeling. Taken together, our results offer necessary understandings of RNF112 as a dynamin-like large GTPase in its cellular function and provide insights into the functional mechanisms of dynamin superfamily proteins.

## Significance

The dynamin superfamily GTPases are key membrane remodelers in cells. As a recently identified member, RNF112 is essential for functional synapses by regulating endosome and mitochondrial morphology, but its mechanisms remain obscure. This study reports the crystal structures of modified RNF112 in different stages of GTP hydrolysis and reveals its unique feature in GTP hydrolysis-coupled domain rearrangement. Based on the structural information, the biochemical properties of RNF112 as a mechanochemical enzyme were systematically investigated and the key residues for its activity were identified. Finally, engineered RNF112 is capable of mediating membrane remodeling in a GTP-dependent manner. These results pave the way for further research characterizing RNF112-related diseases and broaden our understanding of dynamin superfamily.

Ring Finger Protein 112 (RNF112), also known as Zinc Finger Protein 179 (ZNF179) or neurolastin, is a protein that is primarily expressed in the brain (1–3). The gene encoding it is localized within the short arm of chromosome 17 (17p11.2) (4). Deletion of this region can cause a developmental disorder, Smith-Magenis syndrome (SMS), which is characterized by intellectual disability, facial abnormalities, abnormal behavior, and sleep disturbances (4, 5). In mouse models of Huntington’s disease and amyotrophic lateral sclerosis (ALS), RNF112 is downregulated in the motor neurons (6, 7). Recently, an antiapoptotic role of RNF112 was reported in astrocytes in the cortex and hippocampus of the mouse (McGill-R-Thy1-APP) APPtg model of Alzheimer’s disease (8, 9).

A number of studies have elucidated the physiological importance of RNF112 in brain development. In particular, most RNF112 knockout (KO) mice are embryonic lethal, and the surviving mice exhibit growth retardation resulting in smaller size and reduced weight (10). Further investigation has demonstrated that RNF112 KO mice have smaller brains, decreased dendritic spine density, impaired synaptic transmission, and reduced levels of antioxidant enzymes in the hippocampus (11, 12). Brain functions, such as motor balance and spatial learning and memory, are also impaired in RNF112 KO animals (10). Additional roles of RNF112 in the adult brain include the protection of neural tissues from oxidative stress-induced brain injury (13). However, the underlying molecular mechanisms remain elusive.

RNF112 was first identified as a member of the RING finger protein family and acts as an E3 ubiquitin ligase in neuronal differentiation during brain development (14). It contains a classical C3HC4 RING finger domain (referred to as the RING domain). Previous studies have found that RNF112 possesses an autoubiquitination feature and also directly ubiquitinates TDP-43 and Forkhead box M1 (FOXM1) to attenuate related neuropathy and inhibit gastric cancer progression, respectively (15–17). Recently, RNF112 has emerged as an intriguing addition to the dynamin-like large GTPase superfamily (11), which has been widely implicated in mediating biological membrane remodeling events, including membrane tubulation, fission, and fusion (18). Interestingly, at the subcellular level, RNF112 partially localizes to endosomes in steady state and is imported into the mitochondria upon cellular stress (11, 19). These two organelles are smaller in size in neurons from RNF112 KO mice (11, 19). Moreover, the GTP hydrolysis activity of RNF112 is associated with the maintenance of dendritic spine density (19). However, the precise function and mechanism of action of RNF112 as a dynamin-like protein is unknown.

In this study, we address the above questions by presenting the crystal structures of truncated RNF112 in different stages of the GTP turnover cycle. Similar to other dynamin superfamily members, RNF112 undergoes conformational changes during GTP hydrolysis and dimerizes via the GTPase domain in the transition state. However, RNF112 adopts a unique self-restraint conformation in the nucleotide-free and guanosine diphosphate (GDP)-bound states and prefers Mn^2+^ over other divalent cations for GTP hydrolysis. The N-terminal RING domain does not directly affect the above properties of RNF112. We also show that RNF112 can mediate membrane remodeling in vivo in a GTPase-dependent manner. Our findings offer necessary details for understanding the roles of the GTPase activity of RNF112 in its cellular function, and provide insights into the mechanochemical mechanisms of dynamin-like proteins.

## Results

### Overall Structure of RNF112.

**Fig. 1.**
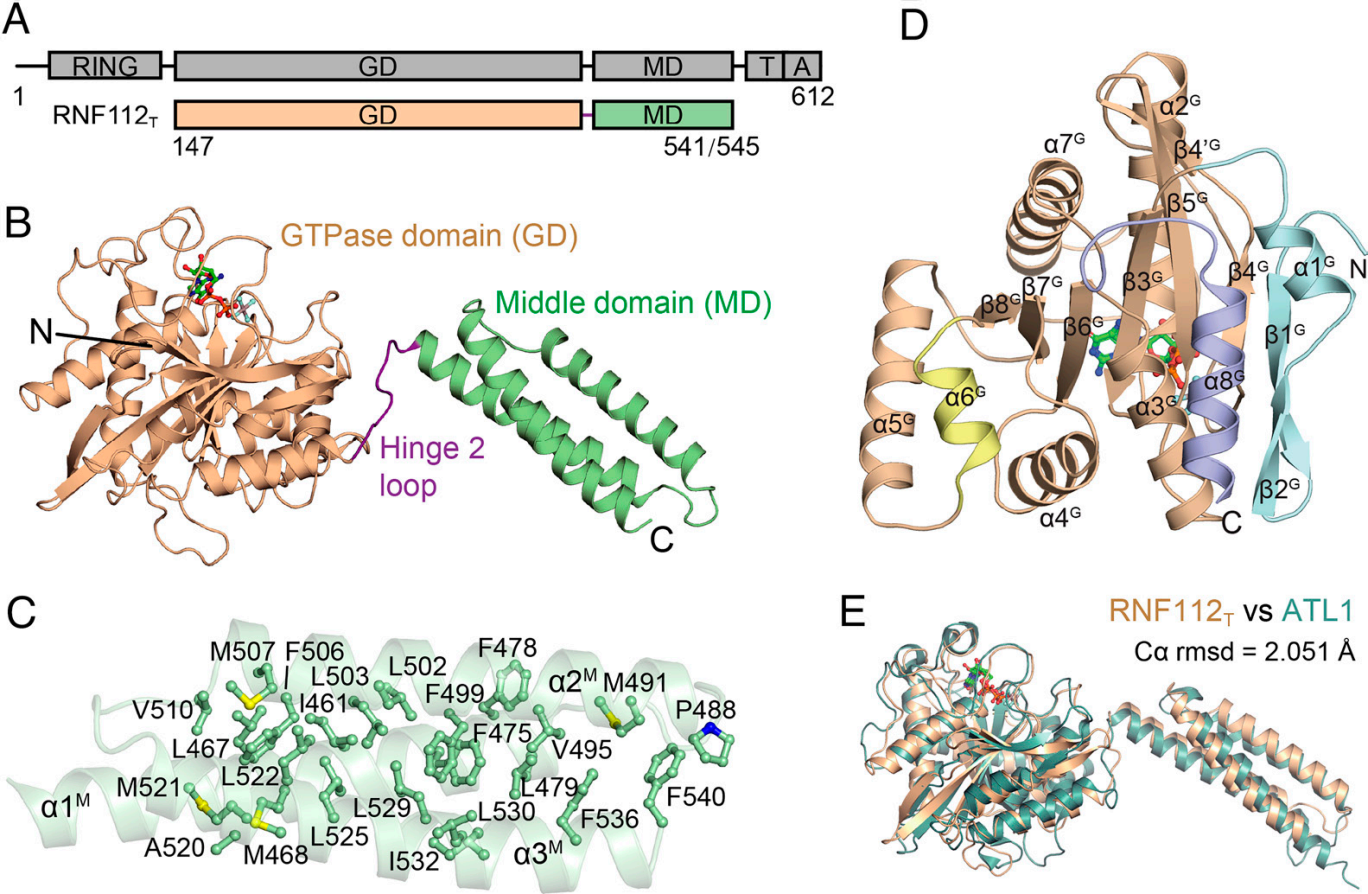
Overall structure of RNF112_T_. (*A*) Schematic representation of the organization of crystallized RNF112T based on full-length xlRNF112. RING, RING domain; GD, GTPase domain; MD, middle domain. Elements for RNF112 are assigned according to the structure. The borders of each element are indicated by residue numbers. (*B*) Structure of RNF112_T_. The domains are indicated and colored as in (*A*). The Hinge 2 loop is in purple. (*C*) Hydrophobic network within the MD. (*D*) The GD of RNF112_T_. α1^G^, β1^G^, β2^G^, α6^G^, and α8^G^ are specified by color. The core region corresponding to Ras is in wheat color. GDP•AlF_4_^−^ is shown as sticks. (*E*) Comparison of the overall structure of RNF112_T_ and ATL1 (Protein Data Bank code: 4IDO).

To obtain an architectural overview of RNF112, we carried out structural studies on modified *Xenopus laevis* RNF112 constructs in different nucleotide-loading states. For these constructs (collectively denoted as RNF112_T_, *SI Appendix*, Fig. S1*A*), the N-terminal RING domain, the predicted transmembrane helix, and the C-terminal amphipathic helix were removed. We crystalized RNF112_T_ in the apo form, in the presence of the hydrolysis-resisting GTP analog GTPγS, or in the presence of GDP•AlF_4_^−^, mimicking the transition state of GTP hydrolysis, and determined their structures at 2.1 Å, 2.2 Å, and 1.9 Å, respectively (*SI Appendix*, Table S1). RNF112_T_ is composed of a GTPase domain (GD) and a middle domain (MD) comprising a 3-helix bundle (Fig. 1 *A* and *B*). The MD is stabilized by hydrophobic interactions within the three bundled α-helices (Fig. 1*C*). Notably, mammalian, reptilian, and some amphibian RNF112 proteins have a longer predicted α1^M^-α2^M^ linker than the *X. laevis* version at the far end of the molecule (*SI Appendix*, Fig. S1*B*). The GTPase domain contains an eight-stranded β-sheet surrounded by eight α-helices. β4^G^ is divided into two portions by a loop formed by the residues from Asn247 to Pro249. Compared to the canonical GTPase Ras, the RNF112 GD has an extra α1^G^, β1^G^, and β2^G^ at the N terminus; α8^G^ at the C-terminus; and a short α6^G^ (Fig. 1*D* and *SI Appendix*, Fig. S1*C*). These inserts have spatially consistent equivalents in other dynamin superfamily members, although some of them are in different position of the primary structure (*SI Appendix*, Fig. S1*D*). Among other dynamin superfamily members, the overall folding of RNF112_T_ resembles that of atlastins (ATLs), and its GD is also topologically related to that of GBP1, another close-relative of ATLs (Fig. 1*E* and *SI Appendix*, Fig. S1*D*). The GD and MD are connected by a linker from Gln448 to Pro455 (Fig. 1*B*), which is analogous to Hinge 2 in other dynamin superfamily members, such as dynamin and mitofusins (20–25).

### Nucleotide Binding and GTP Hydrolysis of RNF112.

**Fig. 2.**
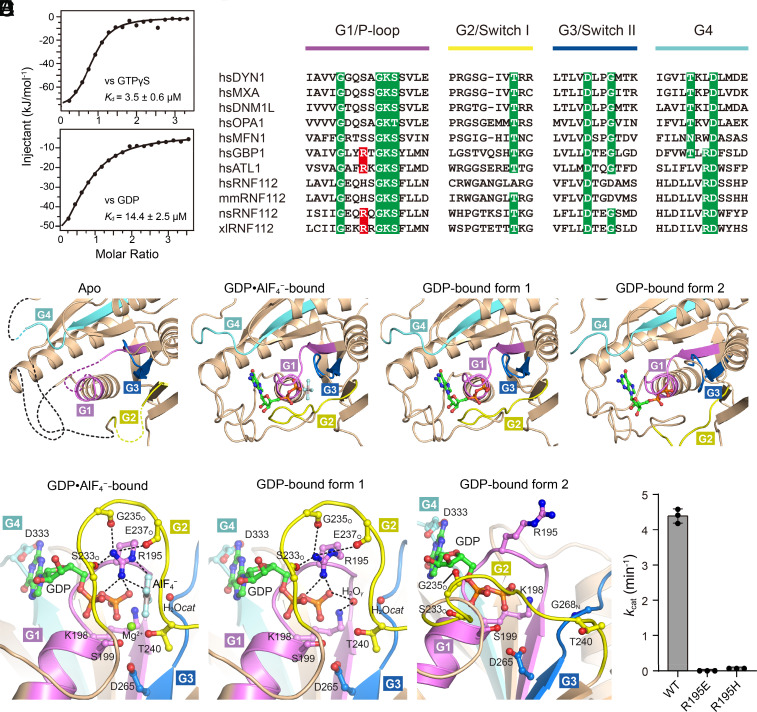
Nucleotide binding and GTP hydrolysis of RNF112_T_. (*A*) Binding affinities of RNF112_T_ for GTPγS and GDP as measured by ITC. The dissociation constants (*K*_d_) are indicated. (*B*) Sequence alignment of the G1–G4 elements for dynamin superfamily members and RNF112 of different species. Conventional motifs/residues are highlighted in green. The key arginine residue for the GTPase activity of RNF112 is highlighted in red. hs, *Homo sapiens;* ns, *Notechis scutatus*; xl, *Xenopus laevis*. (*C*) Structural details of the nucleotide-binding pocket of RNF112_T_ in the nucleotide-free, GDP•AlF_4_^−^-bound, GDP-bound form 1, and GDP-bound form 2 states. (*D*) The GTPase catalytic centers of RNF112_T_ complexed with the indicated nucleotides. The Mg^2+^ ion is shown as a green sphere and water molecules as red spheres. (*E*) The GTP turnover rates of WT RNF112_T_ and RNF112_T_(R195E) and RNF112_T_(R195H). Data are presented as mean ± SD (n = 3).

Next, we investigated the manner of nucleotide manipulation in RNF112. Despite binding GTPγS and GDP with dissociation constants (*K*_d_) in the micromolar range, RNF112_T_ showed no association with the other two GTP analogs, GMPPNP and GMPPCP (Fig. 2*A* and *SI Appendix*, Fig. S2*A*). We also assayed the GTP hydrolysis of RNF112_T_. In a test of different divalent metal ions as coenzymes, we found that the GTPase activity of *X. laevis* RNF112_T_ was highest with the supply of Mn^2+^ at an optimal concentration of 1 mM (*SI Appendix*, Fig. S2 *B* and *C*).

Nucleotide-free RNF112_T_ contains several disordered regions in the nucleotide-binding site in the GD, involving all G1-G4 motifs (Fig. 2 *B* and *C* and *SI Appendix*, Fig. S2*D*), which take up as much as ~15% of the total GD in terms of the number of amino acid residues. The nucleotide-free structure of ATLs is currently unknown, but nucleotide-free GBP1 possesses a well-folded G1 motif/P-loop (26). Thus, the disordered G1 motif of RNF112 in the nucleotide-free state makes it unique in the dynamin superfamily, representing an extremely flexible catalytic center before binding nucleotides. For the RNF112_T_–GTPγS complex, the electron density indicated that the associated guanine nucleotide was actually GDP (*SI Appendix*, Fig. S2*E*), suggesting that GTPγS was eventually hydrolyzed during or after crystallization (hereafter termed RNF112_T_-GDP form 1). In the structure of RNF112_T_-GDP form 1, the G1-G4 motifs are clearly discernable, of which G2/G3 are in the similar catalyzing configurations as in the transition state (Fig. 2*C*). In addition, the crystals of RNF112_T_-GDP form 1 and RNF112_T_-GDP•AlF_4_^−^ bear the same space group, similar unit cell dimensions, and similar molecule packing (*SI Appendix*, Table S1). We also determined another GDP-bound RNF112_T_ structure derived from a nucleotide-free RNF112_T_ crystal soaked with GDP (RNF112_T_-GDP form 2). In this structure, parts of G2/switch I and G3/switch II are disordered, whereas the G1/P-loop and G4 mediating the GDP molecule become discernable compared to the nucleotide-free structure (Fig. 2*C*).

In the GTPase catalytic center of the RNF112_T_-GDP•AlF_4_^−^ complex, the AlF_4_^−^ moiety is closely associated with the β-phosphate group of GDP and coordinated by the catalytic Mg^2+^ ion, a catalytic water molecule (H_2_O*_cat_*), and key residues from G1-G3 motifs (Fig. 2*D*). The side chain of Arg195 stretches toward the catalytic center and intimately contacts the phosphate groups of GDP and the AlF_4_^−^ moiety, and forms hydrogen bonds with the carboxyl oxygens of Ser233, Gly235, and Glu237 of G2/switch I. Arg195 of RNF112_T_ is in an equivalent position of the catalytic Na^+^ ion of dynamin 1 in the transition state (27), and likely to play a role as a domestic arginine finger for the neutralization of the GTP hydrolysis-derived negative charge in a similar manner as ATL1 (28, 29). This residue, while possessed by human ATL1 and GBP1, is not strictly conserved in RNF112 from different species (Fig. 2*B* and *SI Appendix*, Fig. S3). We mutated this arginine to glutamate or histidine as in human and mouse RNF112s, and tested the GTP turnover of the mutants. Compared to wild-type (WT) RNF112_T_, which had a *k*_cat_ of 4.3 min^−1^ at a protein concentration of 20 μM, the GTP turnovers of RNF112_T_(R195E) and RNF112(R195H) were negligible (Fig. 2*E*), suggesting that this arginine finger is indispensable for GTP hydrolysis of RNF112. In contrast, purified human and mouse RNF112_T_ had relatively weak GTPase activity, which could not be rescued by restoring the arginine finger or varying the pH (*SI Appendix*, Fig. S2 *F*–*H*), probably because they lack the canonical key residue(s) in G2 and/or G3 motifs (Fig. 2*B*).

Interestingly, Arg195 of RNF112_T_-GDP form 1, but not of RNF112_T_-GDP form 2, adopts a conformation consistent with that of the RNF112_T_-GDP•AlF_4_^−^ complex at the catalytic site, and maintains the same hydrogens bonds with residues from G2/switch I (Fig. 2*D*). In the structure of RNF112_T_-GDP form 1, a water molecule (H_2_O_γ_) takes up the position of AlF4^−^, and forms hydrogen bonds with the β-phosphate group of GDP, Lys198 of the G1/P-loop, and H_2_O*_cat_*. These interactions led to an atypical-type stabilization of G2/switch I that is analogous to GMPPCP-bound truncated dynamin 1 (30), and rendered RNF112_T_-GDP form 1 crystallized in a transition-like state (similar to RNF112_T_-GDP•AlF_4_^−^) during the slow hydrolysis of GTPγS.

### RNF112 Dimerizes via the GTPase Domain in the Transition State.

**Fig. 3.**
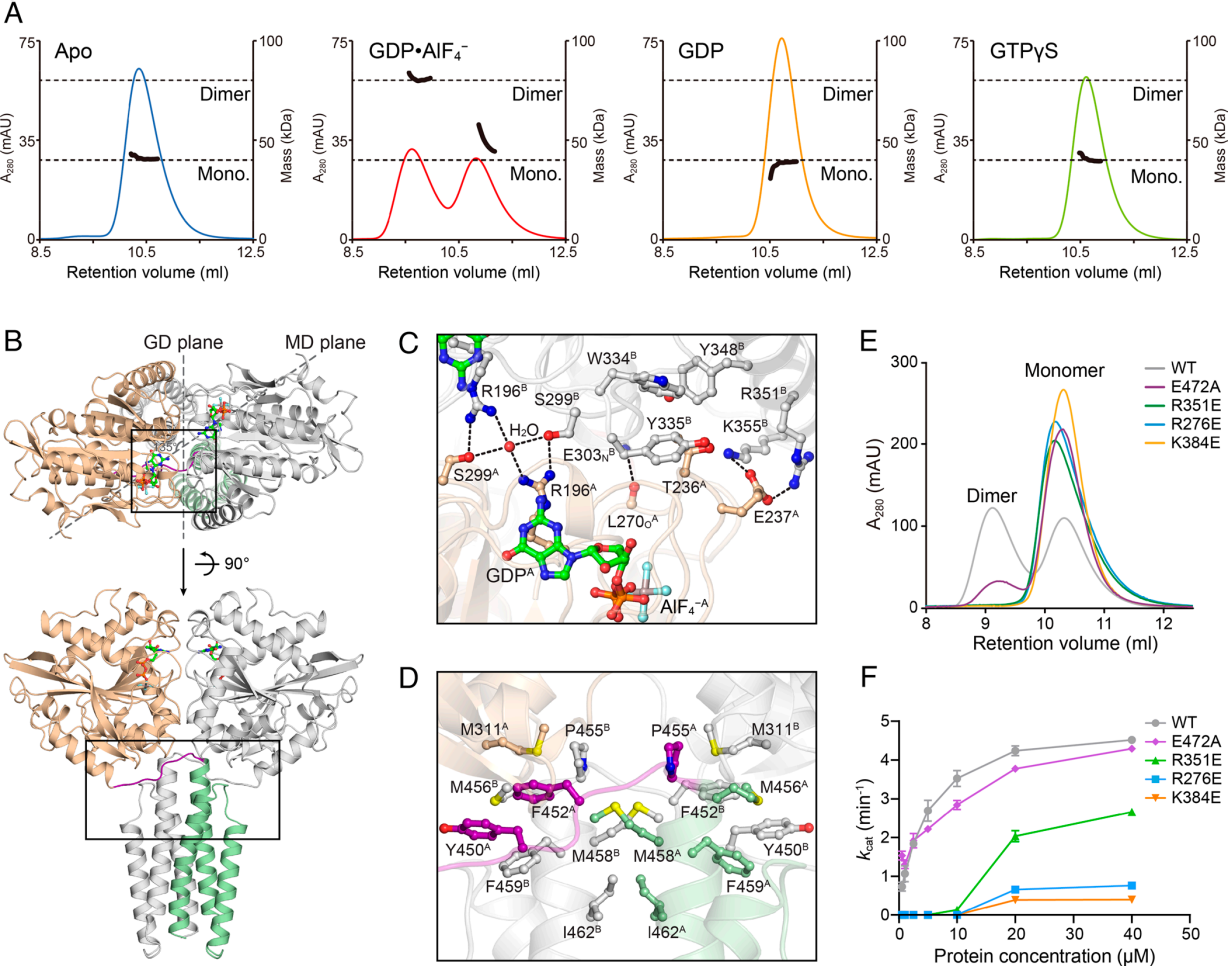
RNF112_T_ dimerizes in the transition state. (*A*) The dimerization properties of RNF112_T_ alone or in the presence of the indicated nucleotides were assayed in analytical gel filtration coupled to RALS. Calculated molecular masses at the absorption peak of 280 nm are plotted in black. mAU, milli-absorption units. (*B*) Structure of RNF112_T_ dimer in the transition state. One protomer is colored as in Fig. 1*B* and the other colored gray. The details of the dimeric interface of the indicated regions are illustrated in (*C*) (upper box) and (*D*) (lower box). (*C*) Details of the intermolecular interaction around the nucleotide-binding site. The side chains of involved residues are shown in the same color as the domains to which they belong. (*D*) Details of the *trans* hydrophobic network around the crossover Hinge 2 loops. (*E*) Dimerization properties of WT RNF112_T_ and the indicated mutants in the presence of GDP•AlF_4_^−^ were assayed in analytical gel filtration. mAU, milli-absorption units. (*F*) GTP turnover rates of WT RNF112_T_ and the indicated mutants were measured at seven different protein concentrations. Data are presented as mean ± SD (n =3).

A canonical feature of dynamin superfamily members is the interdependency of GD homodimerization and GTP hydrolysis (18, 31, 32). According to size exclusion chromatography coupled with right angle light scattering (SEC-RALS) and analytical ultracentrifugation, RNF112_T_ was monomeric in the nucleotide-free, GDP-bound, or GTPγS-bound states in solution, whereas dimers could be formed in the GDP•AlF_4_^−^-bound state (Fig. 3*A* and *SI Appendix*, Fig. S4*A*). In the structure of the RNF112_T_-GDP•AlF_4_^−^ complex, the RNF112_T_ homodimer could be observed within two neighboring asymmetric units of the crystal (*SI Appendix*, Fig. S4*B*). In this dimer, the nucleotide-binding pockets of two GDs sit face-to-face at one end, and the two MDs align parallel at the other, with their C termini pointing in the same direction (Fig. 3*B* and *SI Appendix*, Fig. S4*C*). The two Hinge 2 loops cross each other, resulting in a 135° angle between the bisection planes of the GDs and MDs. The association between the two GDs constitutes the majority of the 3,188 Å^2^ dimeric interface. Although the nucleotides do not directly participate in *trans* contact, the catalytic elements around the nucleotide-binding pocket are deeply involved. In particular, Arg196 on the G1 motif forms a *trans* hydrogen bond with Ser299, Gly235-Glu237 on the G2 motif closely contact the neighboring molecule via salt bridges and hydrophobic interactions, and Leu270 on the G3 motif forms a main-chain hydrogen bond with Glu303 (Fig. 3*C*). Other GD contacts include salt bridges (Glu273-Arg351; Arg276-Glu303/Asp307; and Lys384-Asp398/Asp400), hydrophobic interaction between Ile272 and Leu306/Leu356, and connections via rich solvent molecules (*SI Appendix*, Fig. S4 *D* and *E*). The cross-oriented Hinge 2 loops are also extensively involved in dimer formation by contributing hydrophobic networks with GDs and the close ends of MDs (Fig. 3*D*). In addition, hydrophilic intermolecular contacts are found at the middle and far ends of the MDs (*SI Appendix*, Fig. S4*F*). Consistent with the transition-like structural features as mentioned earlier (Fig. 2*D*), the cross-asymmetric-unit dimerization and above intermolecular residue contacts were also present in the structure of RNF112_T_-GDP form 1 (*SI Appendix*, Fig. S5 *A*–*G*).

We generated single-point mutants for some of these interface residues and checked their dimerization and GTP hydrolysis properties. RNF112_T_(R276E), RNF112_T_(R351E), and RNF112_T_(K384E) all failed to form dimers in solution, and RNF112_T_(E472A) had a declining tendency to dimerize (Fig. 3*E*). WT RNF112_T_ exhibited concentration-dependent GTP turnover, whereas most of the dimeric interface mutants, but not RNF112_T_(E472A), which had preserved partial dimerization potency, showed remarkably weakened GTPase activities (*SI Appendix* and Fig. 3*F*). These results suggest that GD dimerization of RNF112 is required for stimulated GTPase activity.

### RNF112 Exhibits Nucleotide-Dependent Conformational Change.

**Fig. 4.**
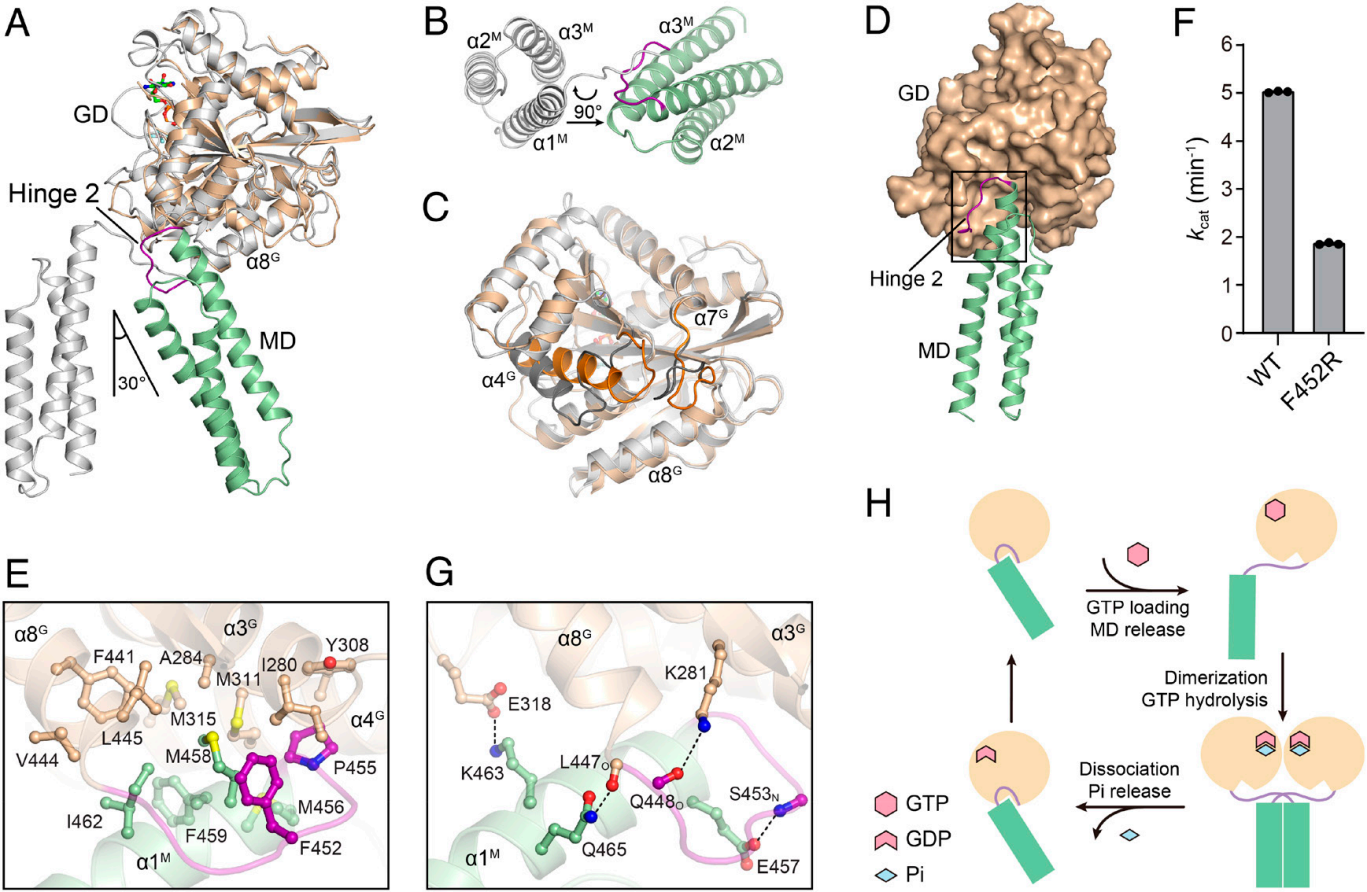
RNF112_T_ exhibits nucleotide-dependent conformational change. (*A*) Structural overlay of nucleotide-free (colored) and GDP•AlF_4_^−^-bound (gray) RNF112_T_ showing the movement of the HD relative to the GD. (*B*) *Top* view of MDs in (*A*). Note the involution of the Hinge 2 loop and the rotation of the MD. (*C*) Comparison of the GDs in (*A*). Note the structural differences specified in dark orange and dark gray for nucleotide-free and GDP•AlF_4_^−^-bound RNF112_T_, respectively. (*D*) The MD wedges into a groove of the GD (shown as surface representation) in nucleotide-free RNF112_T_. Note the backfolded Hinge 2 loop. Details of the boxed area are shown in (*E* and *G*). (*E*) Intramolecular hydrophobic interactions at the GD groove. (*F*) GTP turnover rates of WT RNF112_T_ and RNF112_T_(F452R). Data are presented as mean ± SD (n = 3). (*G*) Extra intramolecular hydrophilic interactions at the GD groove. (*H*) A model of the conformational change in RNF112_T_ during GTP hydrolysis.

Dynamin superfamily members exhibit GTP-hydrolysis-dependent domain movement (20, 24, 25, 28–30, 33–42). For example, the MDs of ATLs take several distinct orientations relative to the GD in the GDP-bound, GMPPNP-bound, and GDP•AlF_4_^−^ states (28, 29, 38, 43). In the RNF112_T_-GDP•AlF_4_^−^ and RNF112_T_-GDP form 1 structures, RNF112_T_ takes a conformation reminiscent of the “tight-parallel” form of ATLs in the GDP•AlF_4_^−^ or GMPPNP-bound states, though the orientations of the MDs exhibit slight displacement (*SI Appendix*, Fig. S6). However, nucleotide-free RNF112_T_ and RNF112_T_-GDP form 2 adopt a unique conformation that has never been observed with ATLs. The MD of nucleotide-free RNF112_T_ stays perpendicular to α8^G^ and swings ~30° from the position of GDP•AlF_4_^−^-bound RNF112_T_ MD when the GDs are superimposed (Fig. 4*A*). Compared to the GDP•AlF_4_^−^-bound form, the Hinge 2 loop in nucleotide-free RNF112_T_ involutes toward the GD from the outstretching conformation, leading to an ~90° rotation around the long axis of the MD (Fig. 4*B*). Importantly, the GD also exhibited local conformational changes at the backside of the nucleotide-binding pocket, involving redirection of helix α4^G^ and relocation of the loop between α7^G^ and α8^G^, which creates a big groove to dock the MD (Fig. 4*C*). Accompanied by the wedging of the MD into this groove are extensive interdomain contacts (Fig. 4*D*). Met456, Met458, Phe459, and Ile462 from the MD; Phe452 and Pro455 from the Hinge 2 loop; and Ile280, Ala284, Tyr308, Met311, Met315, Phe441, Val444, and Leu445 from the GD form a massive intramolecular hydrophobic network (Fig. 4*E*). Notably, many of these residues are responsible for the intermolecular contact in the RNF112_T_-GDP•AlF_4_^−^ dimer (Fig. 3*D*). We performed site-directed mutagenesis for Phe452, a Hinge 2 residue involved in both intramolecular and intermolecular domain contacts for monomeric nucleotide-free RNF112_T_ and dimeric GDP•AlF_4_^−^-bound RNF112_T_, respectively, and applied it to GTP hydrolysis assays. Compared to WT RNF112_T_, the F452R mutant led to a ~2.5-fold decrease of GTPase activity, suggesting that domain contacts mediated by Hinge 2 are critical for the efficiency of GTP hydrolysis (Fig. 4*F*). In addition, a specific salt bridge formed by Glu318 and Lys463 and several hydrogen bonds involving main chain atoms (Ser453_N_-Glu457, Leu447_O_-Gln465, and Gln_O_448-Lys281) are also found in this region (Fig. 4*G*). These residues are conserved within RNF112 from different species, but not ATLs, mitofusions, or yeast Sey1. Due to variances in the Hinge 2 loop and certain regions of the GD, some RNF112 residues have no counterparts in the above dynamin-like membrane fusion proteins (*SI Appendix*, Fig. S3). Therefore, the interdomain contacts are unique to nucleotide-free RNF112 and stabilize its specific conformation (Fig. 4*H*). Last, the “extended” conformation of GDP-bound ATLs (28, 29) was not found for our RNF112_T_ crystal structures.

**Fig. 5.**
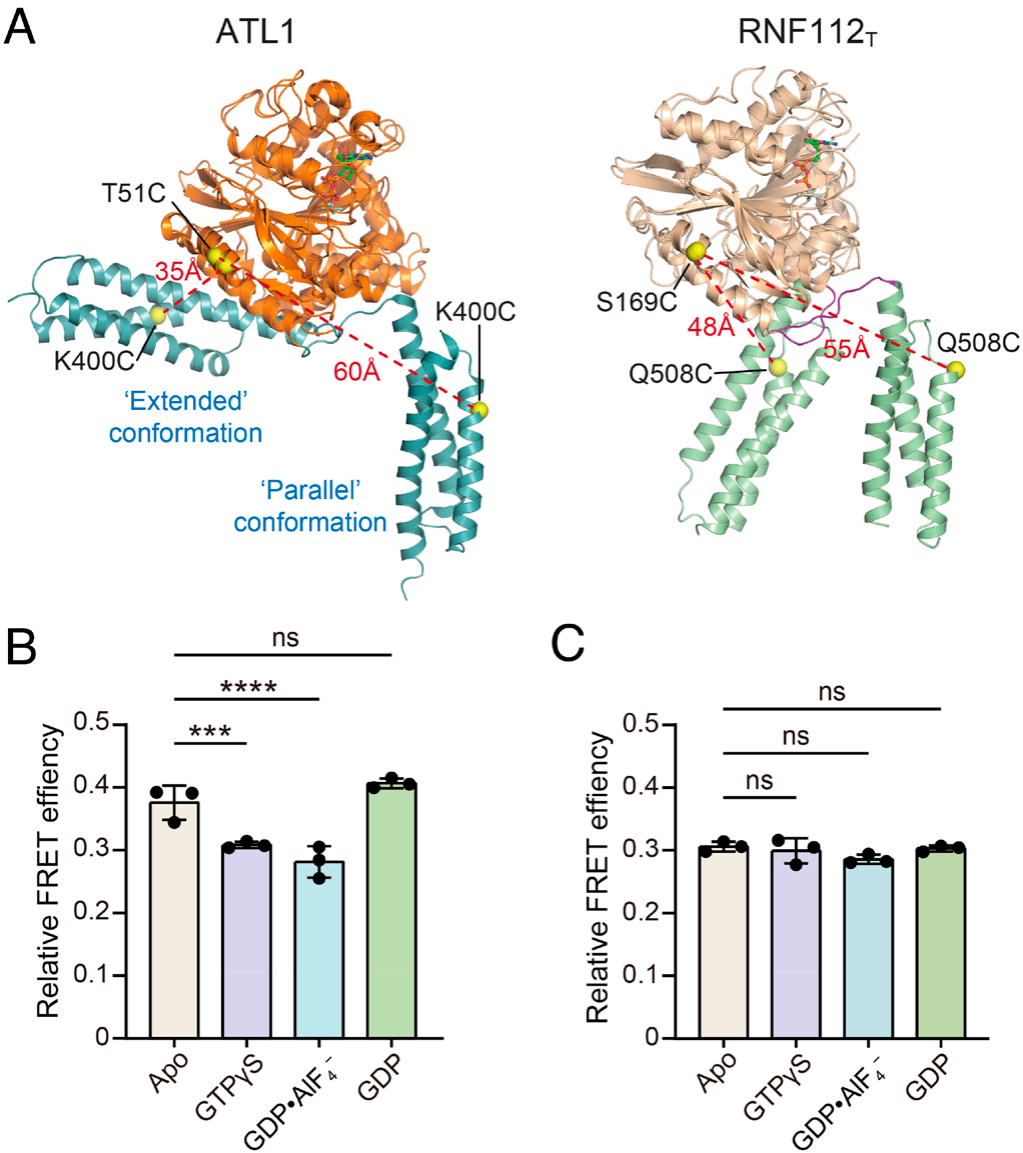
Conformations of RNF112_T_ revealed by intramolecular FRET assays. (*A*) FRET labeling strategy based on the conformational variance in ATL1_cyto_-TK (*Left*) and RNF112_T_-SQ (*Right*) in different nucleotide-loading states. The distances of engineered FRET pairs in each conformation of the two proteins are indicated. The notable variance between the FRET pair distances of the parallel (GDP•AlF_4_^−^-bound, PDB code 4IDO) and extended (GDP-bound form 2, PDB code 3Q5E) conformations of ATL1_cyto_ should incur measurable differences in FRET efficiencies, whereas the relatively little variance between the FRET pair distances of nucleotide-free and GDP•AlF_4_^−^-bound RNF112_T_-SQ molecules may not. (*B* and *C*) FRET efficiency values for ATL1_cyto_-TK (*B*) and RNF112_T_-SQ (*C*) in the absence or presence of the indicated nucleotides. Data are presented as mean ± SD (n = 3). ***P < 0.001; ****P < 0.0001; ns, not significant.

To understand whether RNF112_T_ may adopt an extended conformation like ATL1 during the GTP hydrolysis cycle, we used Förster resonance energy transfer (FRET) to monitor the domain rearrangement in solution. For this assay, we generated a construct of RNF112_T_ in which all native cysteines were substituted with alanines, and residues Ser169/Gln508 from the GD/MD were mutated to cysteines to introduce an intramolecular FRET pair between the two domains (Fig. 5*A* and *SI Appendix*, Fig. S7*A*, termed Cys-less RNF112_T_-S169C/Q508C, or RNF112_T_-SQ). The N-terminal cytosolic region of Cys-less ATL1 carrying T51C/K400C double mutations (ATL1_cyto_-TK), which was generated in our previous single-molecule FRET study on ATL1 (38), was employed as a control. RNF112_T_-SQ and ATL1_cyto_-TK were individually labeled with LD555/LD655 dyes (*SI Appendix*, Fig. S7*B*). To minimize the FRET interference caused by the dimerization of labeled protein during GTP hydrolysis, labeled and unlabeled proteins were mixed at a 1:20 molar ratio to ensure that most of the FRET signals we measured occur within one protomer. For ATL1_cyto_-TK, we observed relatively low bulk FRET efficiency values (~0.3) in the GTPγS- and GDP•AlF_4_^−^-bound states, and high values in (~0.4) the nucleotide-free and GDP-bound states (Fig. 5*B*). According to our previous smFRET results, the low and high FRET efficiency values indicated “parallel” and extended conformations of ATL1_cyto_, respectively (Fig. 5*A*). In contrast, labeled RNF112_T_-SQ exhibited similar FRET efficiency values in different nucleotide-loading states; the values were relatively low compared with ATL1_cyto_-TK in the parallel conformation (Fig. 5*C*). These results suggest that the domain rearrangement of RNF112_T_ is limited in the two conformations observed in our crystal structures at each step of the GTP hydrolysis cycle (Fig. 5*A* and *SI Appendix*, Fig. S6) but does not include the ATL1-like extended conformation.

### Membrane Remodeling Activity of RNF112.

In view of the structural similarity of RNF112_T_ to ATLs, which is a functional ortholog of yeast Sey1p (44–46), we tested whether RNF112_T_ mediates membrane remodeling in *sey1Δyop1Δ* (DKO) yeast cells. Compared with the WT yeast cell, the intricate tubular ER network at the periphery of the DKO cell became much more sparse due to a defect in ER membrane fusion as reported previously (44, 45). To relocate the RNF112 to the ER membrane, we replaced its C-terminal hydrophobic region with the transmembrane domains and C-terminal tail of human ATL1 (RNF112_F__ATL1, *SI Appendix*, Fig. S8*A*). The defects in ER morphology were partially rescued when RNF112_F__ATL1 was expressed (*SI Appendix*, Fig. S8 *A* and *C*). In addition, deletion of the RING domain (RNF112_T__ATL1), but not the GD–MD region (RNF112_R__ATL1), maintained proper ER morphology in DKO cells (*SI Appendix*, Fig. S8*B*). The rescue of defective ER morphology was also obtained when the two transmembrane domains of yeast ER-resident protein Sac1p were added to the C terminus of full-length RNF112 (RNF112_Sac1, *SI Appendix*, Fig. S8 *A* and *C*). The GTPase-deficient mutant (R195E) and another mutant that reduced dimer formation (K384E) were inactive (*SI Appendix*, Fig. S8*C*). Taken together, these data demonstrate that the engineered ER-localized RNF112 is capable of mediating organelle remodeling.

### Autoubiquitination of RNF112 Is Independent of Its Dynamin-Like Properties.

**Fig. 6.**
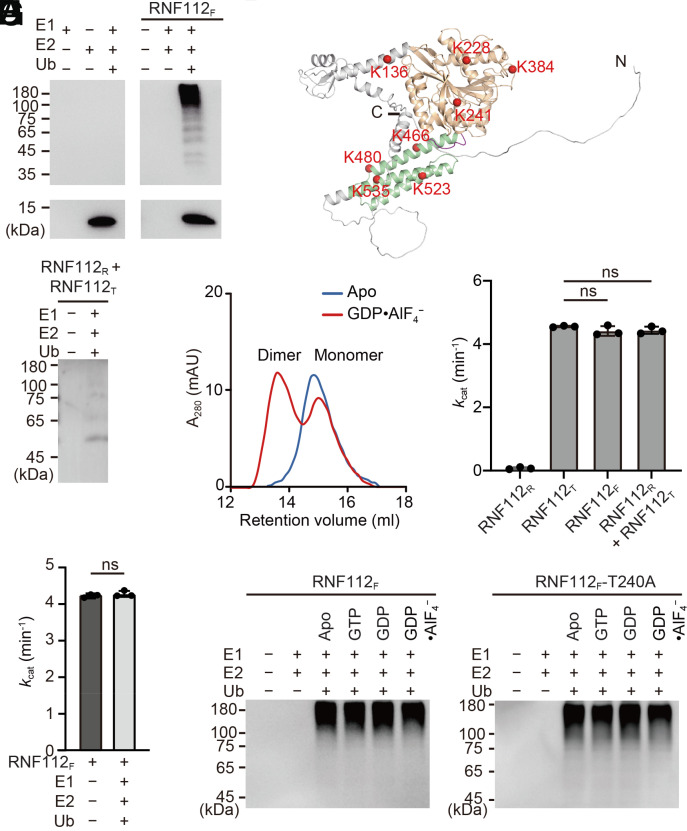
Characterization of the autoubiquitination properties of RNF112. (*A*) In vitro autoubiquitination activity assay. RNF112_F_ was incubated with E1, E2, and ubiquitin in an ATP-regenerating system. The presence of ubiquitin conjugates was determined by immunoblotting with mono-Ub antibody. (*B*) The ubiquitination sites of RNF112_F_ identified by MS are shown as red spheres in the full-length structure predicted by AlphaFold2. Colored regions are included by our crystal structures, and the unresolved regions are shown in gray. (*C*) In vitro ubiquitination assays for RNF112_R_ and RNF112_T_. (*D*) Dimerization of RNF112_F_ in the apo and GDP•AlF_4_^−^-bound forms tested by analytical gel filtration. mAU, milli-absorption units. (*E*) GTP turnover rates of the indicated RNF112 constructs. Data are presented as mean ± SD (n = 3). ns, not significant. (*F*) GTP turnover rates of RNF112_F_ with or without autoubiquitination. Data are presented as mean ± SD (n = 3). ns, not significant. (*G* and *H*) Autoubiquitination of RNF112_F_ (*G*) and RNF112_F_(T240A) (*H*) in the absence or presence of the indicated guanine nucleotides.

Compared to other dynamin superfamily members, RNF112 contains a specific N-terminal RING domain. Though reported to be active, biochemical characterization of the RNF112 RING domain is insufficient, especially to determine whether the dynamin-like portion of RNF112 regulates its E3 ligase activity. Using a purified near-full-length construct containing residues 60 to 541 (termed RNF112_F_; longer versions from the very N terminus of RNF112 turned out to be insoluble, *SI Appendix*, Fig. S9*A*), we tested the autoubiquitination activity of RNF112 with a typical E1 (UBA1) and a reported E2 UBE2D3 (also known as UbcH5c) (11). Polyubiquitin conjugates were clearly observed (Fig. 6*A*), suggesting that RNF112 possesses autoubiquitination activity like reported previously (11). To figure out the exact ubiquitination sites, we performed mass spectrometry (MS) and found eight sites (*SI Appendix*, Fig. S9*B*). Of these sites, seven are located in the GD–MD portion solved in the crystal structure (3 in GD and 4 in MD) and one (Lys136) is on a predicted α-helix linking the RING domain and GD (Fig. 6*B*). These sites are not highly conserved among different species (*SI Appendix*, Fig. S6). Next, we generated a RING domain construct of RNF112 containing residues 68 to 135 (RNF112_R_, *SI Appendix*, Fig. S1*A*) and performed an in vitro ubiquitination assay with RNF112_T_ containing only the GD and MD. Though RNF112_R_ showed no autoubiquitination by itself, we observed very weak polyubiquitin conjugates for the mixture of RNF112_R_ and RNF112_T_ (Fig. 6*C*). These results suggest that direct linkage between the RING domain and ATL-like portion is crucial for the autoubiquitination efficiency of RNF112 and support the finding that ubiquitination sites are located in the ATL-like portion.

By analyzing the positions of these ubiquitination sites on the RNF112_T_ structure, we found that some residues are involved or buried in the dimeric interface (Lys384, Lys480, and Lys535) (*SI Appendix* and Fig. 4 *D* and *F*). We then investigated whether the dynamin superfamily properties and E3 ligase activity of RNF112 have a mutual impact on each other. RNF112_F_ became dimerized in the presence of GDP•AlF_4_^−^ and showed similar GTPase activity as RNF112_T_ (Fig. 6*E* and *SI Appendix*, Fig. S6*D*). Addition of the RING domain did not alter the GTP turnover of RNF112_T_ (Fig. 6*E*). Furthermore, autoubiquitination of RNF112_F_ did not alter its GTPase activity (Fig. 6*F*). These results suggest that the GTP hydrolysis machinery is not directly influenced by the autoubiquitination function of RNF112. We then tested the autoubiquitination of RNF112_F_ in the presence of different guanine nucleotides. In the presence of GTP, GDP, or GDP•AlF_4_^−^, RNF112_F_ exhibited similar autoubiquitination efficiency as in the nucleotide-free state (*SI Appendix*, Fig. S6*G*). We also performed autoubiquitination assays on the GTPase-deficient mutant RNF112_F_(T240A), which bound GTP but did not dimerize (*SI Appendix*, Fig. S9 *C* and *D*), and the dimeric interface (also one of the ubiquitination sites) mutant RNF112_F_(K384E). Both mutants showed similar autoubiquitination efficiencies in different nucleotide-loading states (Fig. 6*H* and *SI Appendix*, Fig. S9*E*). These data demonstrate that the autoubiquitination function of RNF112 is independent of its GTPase activity, as well as the associated dimerization and domain rearrangements.

## Discussion

Our investigation of RNF112_T_ uncovered its resemblance as well as unique features in comparison with other members of the dynamin superfamily proteins, including its close relatives ATL1 and GBP1. The crystal structures of RNF112_T_ in different nucleotide-bound states prove the molecular basis underlying the GD dimerization-stimulated GTPase activity of a unique dynamin-like protein. In summary, Arg196 and Gly235-Glu237 on the catalytic elements that mediate contact between the two GDs were missing from the nucleotide-free structure, indicating that the dimerization of GDs likely contributes to the stabilization of the G1/G2 motifs at the catalyzing configurations, thereby stimulating GTP hydrolysis, which is a typical mechanism of the dynamin superfamily members and reported also for other multidomain GTPases such as MnmE and FeoB (47, 48). Furthermore, when in complex with GDP•AlF_4_^−^, the structure of RNF112_T_ adopts an ATL1-like tight crossover dimer conformation though with a slight rotation of the tightly associated MDs.

Given the similarities with other dynamin superfamily members, a special structural feature of RNF112 is seen at its nucleotide-free state, which is characterized by a 30° rigid-body swing of the MD relative to the GD and a new intramolecular interface between the two domains. This intramolecular interaction is likely to be unique to RNF112, as the key residues involved are not conserved with ATLs. This unique MD–GD docking conformation of RNF112 may explain why it has much lower GTPase activity than ATL1 (43), as the large-scale conformational change against extensive hydrophobic intramolecular associations (shown in Fig. 4*E*) would consume extra time during GTP hydrolysis cycle. In addition, unlike ATLs, our FRET-based biochemical analysis confirmed that RNF112 does not adopt an extended conformation during the GTPase cycle. Thus, our structural and biochemical results lead to a comprehensive model of the conformational dynamics of RNF112 coupled with GTP hydrolysis (Fig. 4*H*). Finally, the RING domain does not directly participate in protein dimerization, and the E3 ligase and GTPase activity of RNF112 are unrelated to each other, though efficient self-ubiquitination requires the RING domain and GD–MD region to be in the same polypeptide chain.

Sequence alignments and structure comparisons of RNF112_T_ with other dynamin-like proteins revealed that it is most closely related to the ER membrane fusion protein ATLs. Demonstration of the fusion activities of most membrane fusogens relies on the liposome-based in vitro lipid-mixing assays using purified proteins. However, some of them lack fusion activities in vitro. For example, although human ATLs function in the cells, they have not been demonstrated to induce proteoliposome fusion in vitro (49, 50) until recently, when the purification system and the lipid compositions of the liposomes were optimized (51–53). Thus, it is tricky to reconstitute protein-mediated membrane fusion in vitro. Alternatively, reexpression of human ATL1, its plant ortholog RHD3, or MFN1-ATL1 chimeras that were artificially relocated to the ER are able to replace ER fusogen Sey1p and restore the ER morphology in *sey1Δyop1Δ* yeast cells (44, 54–56). This is a well-established system for testing the fusion activities of dynamin-like membrane fusion proteins in vivo. Using this strategy, we found that RNF112 can mediate ER membrane fusion in yeast when we relocate it to the ER using the transmembrane domains and C-terminal tail of human ATL1 or the transmembrane domains of yeast Sac1p. Given that RNF112 partially colocalizes with the endosomes and translocates to mitochondria upon cellular stress in brain tissue (11, 19), our evidence suggests that, in addition to mediating protein ubiquitination in the control of protein homeostasis, RNF112 also acts as a stress-responsive fusogen to regulate organelle dynamics in neurons. Consistent with this, more fragmented mitochondria were observed in the RNF112 KO mice (19). The relationship between RNF112 and other well-known membrane fusion proteins in these organelles, including SNARE complex, MFN1/2, and OPA1, in brain development is yet to be discovered.

Our structural and biochemical analyses also provide further mechanistic insights into membrane fusion catalyzed by RNF112, which could be shared with other dynamin superfamily proteins, such as ATLs and MFNs. First, the dimerization of RNF112 molecules, which is a prerequisite for pulling the opposing membranes together, only occurs in the presence of GDP•AlF_4_^−^, confirming that GTP hydrolysis, but not GTP binding, directly drives membrane fusion. Second, the domain rearrangements coupled with the GTPase cycle allow protein recycling, but the degree of the rigid-body swing of the MD may not be critical for these steps. Each fusion-specific dynamin-like protein may adopt a unique conformation for loading and/or releasing nucleotide, and the physiological significance deserves further investigation.

## Materials and Methods

### Molecular Cloning.

The cDNA for *X. laevis* RNF112 (xlRNF112) (NCBI accession number NC_054387.1) was purchased from GenScript Biotech Corporation. The cDNAs of truncated constructs, including those for crystallization and the indicated mutants for biochemical assays, were individually cloned into the pSUMO vector with an N-terminal tandem His_6_-SUMO-tag followed by a Ubl-specific protease 1 (ULP1) cleavage site. For the in vitro E3-ligase autoubiquitination assay, the RING domain of xlRNF112 (RNF112_R_) was cloned into pGEX-6p-1 vector with an N-terminal glutathione S-transferase (GST) tag. The details of major constructs are illustrated in *SI Appendix*, Fig. S1*A*. The indicated mutants were prepared by site-directed mutagenesis based on corresponding recombinant plasmids and confirmed by sequencing.

### Protein Expression and Purification.

The recombinant proteins were expressed in Rosetta (DE3) *Escherichia coli* cells and purified using the buffer described previously (57). Transformed bacteria were grown at 37 °C to an OD600 of 0.6 before induction with 0.1 mM isopropyl-1-thio-β-D-galactopyranoside (IPTG) overnight at approximately 17 to 18 °C. For the RNF112_T_ and RNF112_F_ constructs, cells were harvested and lysed by high-pressure homogenization in buffer A [50 mM HEPES (pH 7.0), 400 mM NaCl, 30 mM imidazole, 1 μM DNase I, 1 mM phenylmethanesulfonylfluoride (PMSF), and 2.5 mM β-mercaptoethanol (β-ME)] and were clarified by centrifugation at 40,000 g for 1 h. The target protein was first purified by a Ni-NTA column (Cytiva), and the His_6_-SUMO-tag was removed by His-tagged ULP1. After cleavage, the protein was dialyzed overnight against buffer B [20 mM HEPES (pH 7.0), 400 mM NaCl, and 2.5 mM β-ME]. The target protein was reisolated by a second Ni-NTA column to remove ULP1. Cells overexpressing GST-tagged RNF112_R_ were harvested and lysed in buffer C [20 mM HEPES (pH 7.0), 400 mM NaCl, and 2.5 mM β-ME]. The target protein was purified by a similar procedure to the RNF112_T_ and RNF112_F_ but using a GST column (Cytiva) and the GST tag was cleaved by GST-fused PreScission protease (PSP). All proteins were further purified by size exclusion chromatography on a Superdex 75 16/60 column (Cytiva) or Superdex 200 16/60 column(Cytiva) in buffer containing 20 mM HEPES (pH 7.0), 150 mM NaCl, and 1 mM dithiothreitol (DTT).

### Protein Crystallization.

Purified xlRNF112 constructs were preincubated with the corresponding nucleotides at 10-fold concentration relative to protein for 2 h before being crystallized at 18 °C via hanging drop vapor diffusion by mixing equal volumes of protein (approximately 10 to 15 mg/mL) and reservoir solution. Crystals of apo RNF112_T_ grew from 2% Tacsimate (pH 6.0) and 14 to 16% PEG 3350 overnight. These crystals were then soaked in reservoir solution mixed with an equal volume of gel filtration buffer supplemented with 20% glycerol for cryoprotection. GDP-bound RNF112_T_ grew in 41% Tacsimate (pH 7.0) overnight in the presence of 10-fold GTPγS and cryoprotected by reservoir solution containing an extra 20% glycerol. Crystals of RNF112_T_ in the transition state were obtained in 8% Tacsimate (pH 7.0) and 20% PEG 3350 in the presence of 10-fold GDP, 10-fold AlCl_3_, and 100-fold NaF and directly flash-frozen in liquid nitrogen. For the GDP-bound form 2 structure, crystals of apo RNF112_T_ were transferred to soaking liquid containing 2% Tacsimate (pH 6.0), 15% PEG 3350, and 10-fold GDP for incubation for 1 d at 18 °C. Crystals were directly flash-frozen in liquid nitrogen. All crystals were stored in liquid nitrogen before performing diffraction assays.

### Structure Determination.

Most diffraction datasets were collected at beamlines BL02U1 and BL19U1 of the Shanghai Synchrotron Radiation Facility (SSRF) (58, 59). Datasets were processed using the XDS suite (60). Initial phases were obtained by molecular replacement with a search model generated by AlphaFold2 (61) and refined using Phaser (62). Initial models were built with COOT (63) and refined with PHENIX (64). Structural validation was carried out using MolProbity (65). Structural illustrations were prepared using the PyMOL Molecular Graphic Systems (version 2.5.2, Schrödinger LLC; https://www.pymol.org/).

### RALS.

A coupled RALS-refractive index detector (Malvern) was connected in line to an analytical gel filtration column (Superdex 200 10/300, Cytiva) to determine the absolute molecular masses of the applied protein samples. For each experiment, 25 μM purified RNF112_T_ (WT or mutants) was incubated with or without 1 mM of the corresponding ligand for 6 h at 25 °C before performing measurements. The column was equilibrated with 20 mM HEPES (pH 7.0), 30 mM NaCl, and 1 mM DTT. Data were analyzed using the provided OMNISEC software. All experiments were repeated at least twice, and the data showed satisfying consistency.

### GTP Hydrolysis Assay.

Four divalent cations, including Mg^2+^, Mn^2+^, Ca^2+^, and Zn^2+^ were analyzed as cofactors of GTP hydrolysis. GTP hydrolysis assays for RNF112_F_, RNF112_T_, RNF112_R_, and corresponding mutants were carried out at 37 °C in 20 mM HEPES (pH 7.0), 150 mM NaCl, 1 mM MnCl_2_, and 1 mM DTT as described earlier. For measuring the stimulated GTP turnover of WT RNF112_T_, RNF112_T_(R276E), RNF112_T_(R351E), and RNF112_T_(R384E), proteins at concentrations of 0.5, 1, 2.5, 5, 10, 20, and 40 μM were individually mixed with 1 to 2 mM GTP and the hydrolysis rates determined from a linear fit to the initial rate of the reaction (<40% GTP hydrolyzed). For other experiments, 20 μM protein and 1 mM GTP were used.

### Nucleotide-Binding Study.

The equilibrium dissociation constants for RNF112_T_ and indicated mutants of guanine nucleotides were determined by isothermal titration calorimetry (ITC) using a MicroCal PEAQ-ITC (MicroCal Technology) at 25 °C. The nucleotide (300 µM) was titrated in 2 μL steps against 30 μM protein in buffer containing 20 mM HEPES (pH 7.4), 150 mM NaCl, 5 mM MgCl_2_, and 1 mM DTT. The titration data were then analyzed by MicroCal PEAQ-ITC Analysis Software v1.41. All experiments were repeated at least twice, and the data showed satisfying consistency.

### Analytical Ultracentrifugation.

Sedimentation velocity experiments were performed using an Optima Analytical Ultracentrifugation (AUC) (Beckman Coulter). The proteins (0.03 mM) with or without the indicated nucleotides were prepared in buffer containing 20 mM HEPES (pH 7.4), 150 mM NaCl, 5 mM MgCl_2_, and 1 mM DTT. All interference data were collected at 12 °C at a speed of 40,000 rpm using an An-50 Ti rotor. Data were analyzed using the continuous c(s) size distribution model in the program SEDFIT.

### FRET.

The proteins were incubated with LD555 and LD655 at a ratio of 1:1.2:1.2 in labeling buffer (25 mM HEPES, pH 7.4, 150 mM KCl, 5 mM MgCl_2_) for 5 h at 4 °C. The fluorescently labeled protein and unlabeled protein were mixed 1:20 in the absence or presence of the indicated nucleotide for 1 h at 4 °C. The FRET assays were performed in 100 μL volumes using a Cytation 5 Imaging Reader (BioTek). The donor LD555 was excited at 522 nm, and the fluorescence intensities of LD555 and acceptor LD655 were monitored using an emission wavelength of 575 nm and 685 nm, respectively. The FRET efficiency was calculated using the following equation: FRET = I_A_/(I_D_ + I_A_), where I_D_ and I_A_ represent the LD555 and LD655 fluorescence intensities, respectively.

### Fluorescence Microscopy in Yeast.

Yeast strains used in this study were BY4741 (MATa his3Δ1 leu2Δ2met15Δ ura3Δ) and JHY4 (BY4741 sey1Δ::kanMX4 yop1Δ::HIS3MX6) (45). The plasmid pWP1098, a LEU2/CEN plasmid encoding ER marker Sec63p-GFP, was described previously (45). The chimeras expressed in yeast were obtained by PCR with overlapping primers and subcloned into the pESC-URA vector using the BamHI and SalI sites. Yeast cells were transformed using the lithium acetate method and grown in appropriate synthetic medium to an optical density at 600 nm (OD600) of 1.2 to 1.5. All images were acquired with a superresolution microscope (DeltaVision OMX SR, Cytiva) with a 60× oil-immersion objective. The images were captured by a scientific complementary metal oxide semiconductor (sCMOS) camera and analyzed using ImageJ.

### MS.

All data were collected using a Q-Exactive HF-X equipped with an Easy-nLC 1200 chromatography system (Thermo Fisher Scientific). The nanoLC separation was performed at a flow rate of 250 nL/min using an integrated spraytip column (20 cm × 100 μm i.d) packed with C4 (3 μm/120 Å, 0.5 to 0.8 cm, Maisch GmbH) and C18 (1.9 μm/120 Å, 19 to 20 cm, Maisch GmbH). Peptides were separated using a binary buffer system of 0.1% FA (v/v) in water (buffer A) and 0.1% (v/v) FA in ACN (buffer B). Peptides were separated in an 80 min segmented gradient as follows: 4 to 8% (v/v) buffer B in 2 min, 8 to 28% (v/v) buffer B in 55 min, 28 to 40% (v/v) buffer B in 5 min, 40 to 97% (v/v) buffer B in 2 min, followed by a 16 min wash with 90% (v/v) buffer B. Full MS scans were acquired from m/z 350 to 1,550 with a mass resolution of 120,000. The resolved fragments were scanned at a mass resolution of 15,000 and AGC target value of 1E5. The top 30 parent ions with a charge of 2 or higher were fragmented by higher energy collisional dissociation (HCD) fragmentation. Ubiquitinated peptides were fragmented by HCD with a normalized collision energy (NCE) of 27. The isolation window was 1.4 Da, and the dynamic exclusion time was 30 s with a max injection time of 50 ms. Raw files were searched using MaxQuant (version 1.6.14) with the UniProtKB *X. laevis* database (downloaded from UniProt on 29 July 2024) for the database search. Oxidation (M), deamidation (N), and GlyGly (K) were set as variable modifications, whereas carbamidomethyl (C) was a fixed modification. The PSM and protein FDR were set at 0.01 for identification.

### In Vitro Autoubiquitination Assay.

Ubiquitination reaction mixtures were prepared in a total volume of 30 μL, consisting of 40 mM Tris-HCl (pH 7.0), 5 mM CaCl_2_, 2 mM ATP, 2 mM DTT, 50 ng E1 (UBE1), 250 ng E2 (UBE2D3), 1 μg ubiquitin, and 500 ng of purified protein. The mixtures were incubated at 37 °C with agitation for 1 h by placing Eppendorf tubes containing the reaction mixture on a benchtop tube rotator. 5× SDS sample buffer was added to the mixtures to terminate the reaction and proteins detected by western blotting.

### Western Blot Analysis.

The following antibodies were used for western blot assays in this paper: anti-ubiquitin antibody (1:1,000) and HRP-conjugated anti-mouse secondary antibody (Cell Signaling Technology, 7076, 1:10,000 dilution). The reaction samples were separated by 12.5% SDS-PAGE in 1× SDS running buffer at 120 V for 150 min and transferred to PVDF membranes (Thermo Scientific) in 1× transfer buffer at 220 mA for 2 h. After 1 h incubation with blocking buffer (5% BSA), the membrane was incubated with the corresponding antibodies overnight at 4 °C. The membrane was washed and incubated with HRP-conjugated anti-mouse secondary antibodies. The membranes were washed again, developed with ECL Western Blotting Detection Reagents (Thermo Scientific), and visualized using the ChemiDoc™ Touch Imaging System (Bio-Rad).

## Supplementary Material

Appendix 01 (PDF)

## Data Availability

The X-ray crystallographic coordinates and structure factor files for the RNF112_T_ structures have been deposited in the Protein Data Bank (PDB) under the following accession numbers: 9JJU (apo) (66), 9JJV (GDP•AlF_4_^−^-bound) (67), 9JJW (GDP-bound form 1) (68), and 9JJX (GDP-bound form 2) (69). All other data are included in the manuscript and/or *SI Appendix*.
