# Supplementary material for: Structural and functional characterization of the brain-specific dynamin superfamily member RNF112
Source: Proc Natl Acad Sci U S A. 2025 Apr 8;122(15):e2419449122. doi: 10.1073/pnas.2419449122 (PMC12012479; doi:10.1073/pnas.2419449122)
Supplement: Supplementary file 1 — Appendix 01 (PDF) [file pnas.2419449122.sapp.pdf]

PNAS

Supporting Information for
Structural and functional characterization of the brain-specific dynamin-superfamily member RNF112

Ya-Ting Zhong[1,7], Li-Li Huang[1,7], Kangning Li[2,7], Bingke Yang[2], Xueting Ye[3], Hao-Ran Zhong[1], Bing Yu[1], Menghan Ma[2], Yuerong Yuan[4], Yang Meng[4], Runfeng Pan[4], Haiqing Zhang[4], Lijun Shi[2], Yunyun Wang[2], Ruijun Tian[3], Song Gao[1,5,*], Xin Bian[2,6,*]

[7]These authors contributed equally: Ya-Ting Zhong, Li-Li Huang and Kangning Li

*Corresponding author: Song Gao and Xin Bian
Email: gaosong@sysucc.org.cn and xin.bian@nankai.edu.cn

This PDF file includes:

Figures S1 to S9
Table S1

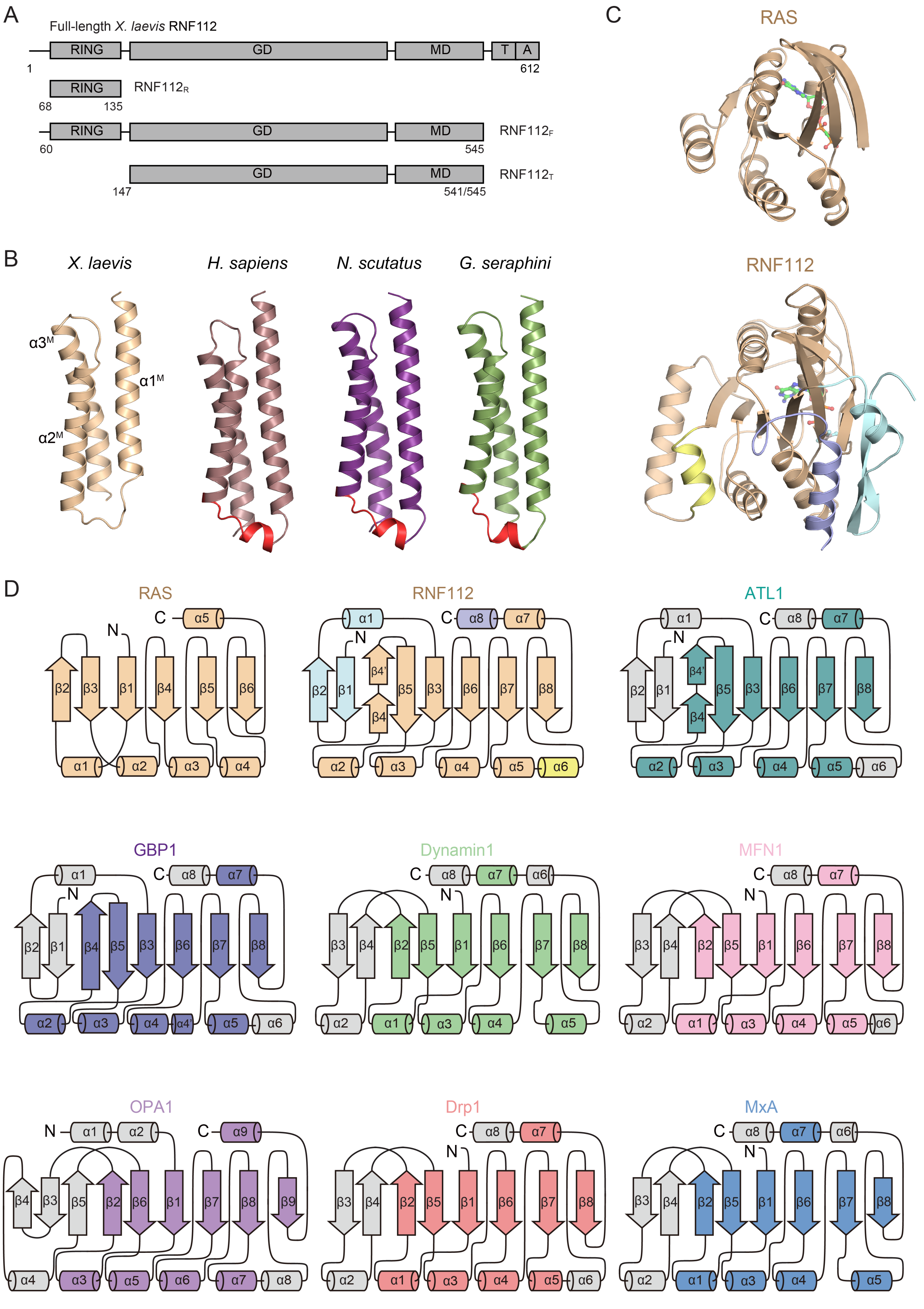

Fig. S1. RNF112 constructs and overall structure of RNF112$_T$.

(A) Schematic representation of the strategy for generating *Xenopus laevis* RNF112 constructs for

crystallization. The labels and numbers are the same as in **Fig. 1*A***.

(B) The MDs of RNF112 from *Xenopus laevis* (UniProt accession Q2VPQ0), *Homo sapiens* (Q9ULX5), *Notechis scutatus* (A0A6J1VXM5), and *Geotrypetes seraphini* (A0A6P8RFL9) predicted by AlphaFold2. Note the longer $\alpha1_M$–$\alpha2_M$ linkers at the far end of RNF112 from *H. sapiens*, *N. scutatus*, and *G. seraphini*, which are colored in red.

(C) The GD of RAS (Protein Data Bank code: 121p) and $RNF112_T$. The core region corresponding to Ras is in wheat color.

(D) Topology diagrams of the G domains of RAS, RNF112, ATL1, GBP1, Dynamin1, MFN1, OPA1, Drp1 and MxA. Secondary structural elements were not drawn to scale.

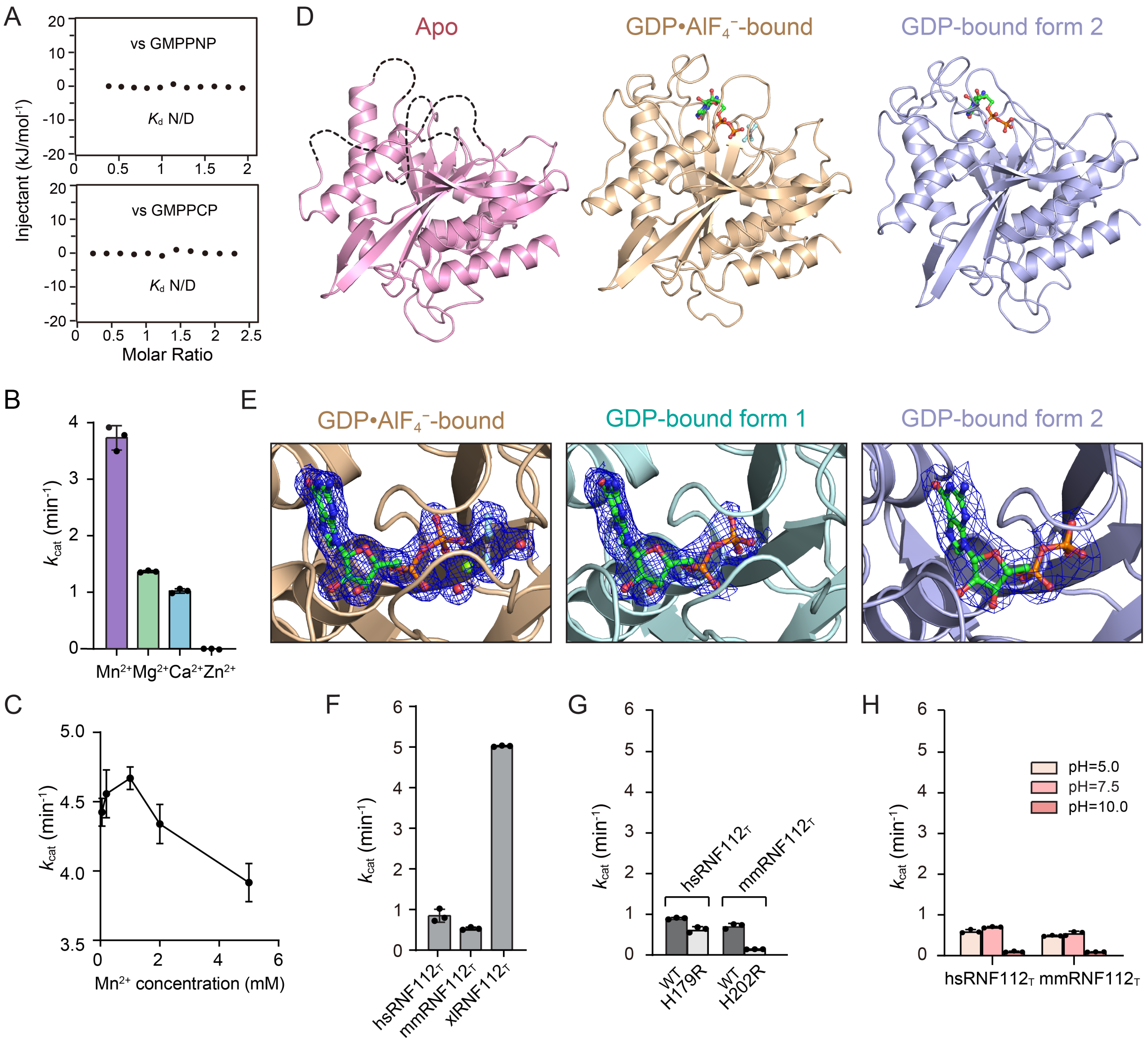

Fig. S2. Nucleotide binding and GTP hydrolysis of RNF112$_T$.

(A) Binding affinities of RNF112$_T$ for GMPPNP and GMPPCP as measured by isothermal titration calorimetry (ITC).

(B) The GDs of RNF112$_T$ in the indicated nucleotide-loading states. Nucleotides are shown as ball-and-stick models. Note the massive disordered regions of RNF112$_T$ in the apo state.

(C) Electron density of the guanine nucleotides in the structure of RNF112$_T$ in the indicated nucleotide-loading states. The electron density maps are shown as blue meshes at a contour level of 1.2σ and nucleotides as ball-and-stick models.

(D) Test of divalent cations as cofactors of RNF112$_T$ for GTP hydrolysis. 20 μM RNF112$_T$ was mixed with 2 mM GTP and 2 mM of each of the divalent cations.

(E) Analysis of the optimal concentration of Mn^{2+} ion for GTP hydrolysis by RNF112$_T$. Data are presented as mean ± SD (n = 3).

(F) Comparison of the GTP turnover rates of *X. laevis* RNF112$_T$ and mammalian RNF112$_T$. Data are presented as mean ± SD (n = 3).

(G) Comparison of the GTP turnover rates of hsRNF112$_T$ (WT and H179R) and mmRNF112$_T$ (WT and H202R). Data are presented as mean ± SD (n = 3).

(H) Comparison of the GTP turnover rates of hsRNF112$_T$ and mmRNF112$_T$ at different pH values.

Data are presented as mean ± SD (n = 3).

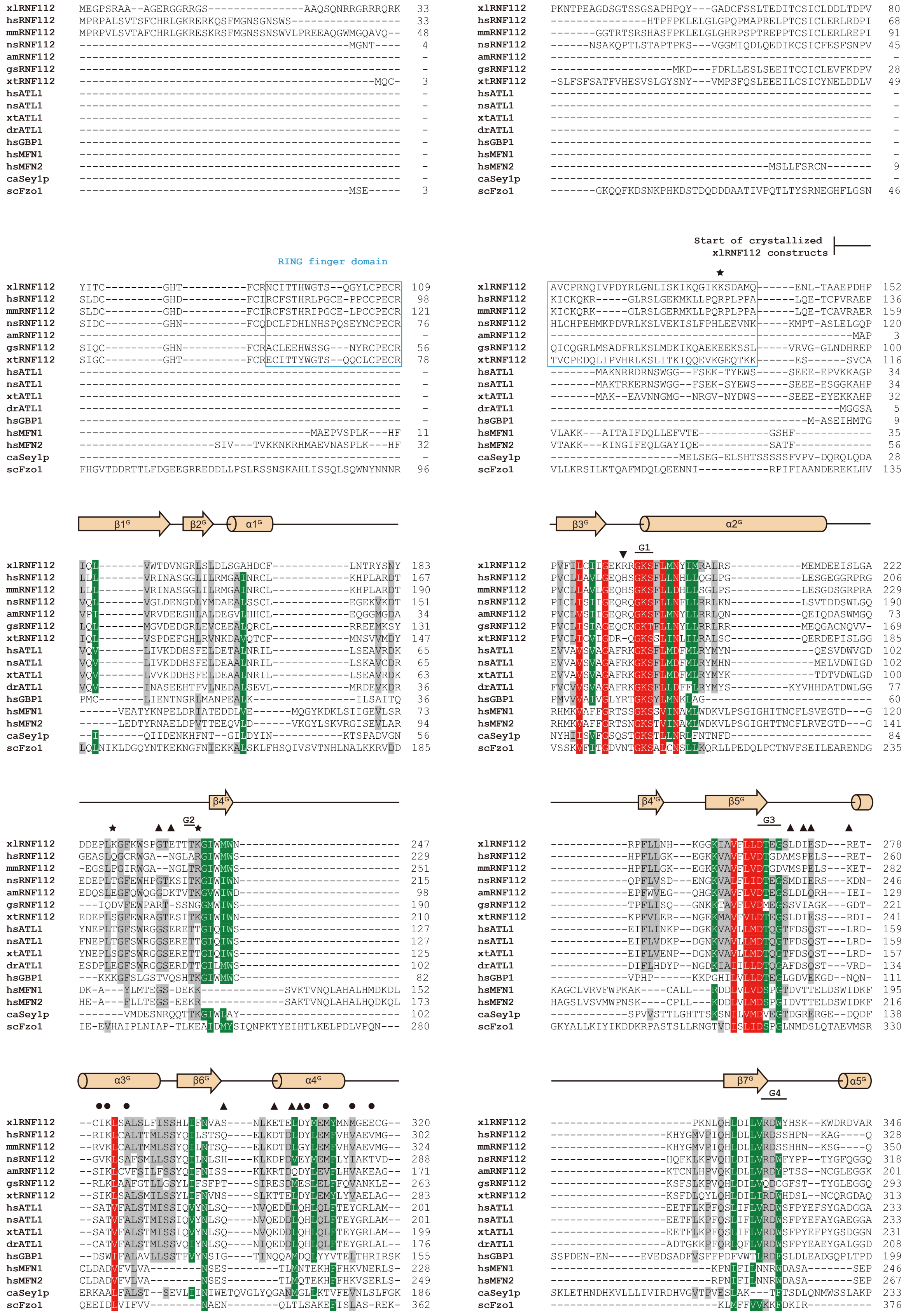
RING finger domain
Start of crystallized xlRNF112 constructs
G1
G2
G3
G4

```
α5G  α6G  β8G
xlRNF112  SYISREI------------EKLEK--LNSYPKVLW-S-----LKSNQTRC  376
hsRNF112  GHVGNIF------------QRLSG----RYPKVQE-L-----LQGKRARC  356
mmRNF112  GHVGDIL------------QKLSG----KYPKVQE-L-----LLGKRARC  378
nsRNF112  VYLHDII------------QKLEG-QPNRHSQALQ-M-----FKSTNTSC  349
amRNF112  RYLSYVI------------QELEA--ASDGSLALE-T-----LR--KARC  229
gsRNF112  EFLRDIM------------QKLQM--CSKYPRTLE-M-----LKYK-SRC  322
xtRNF112  SYINRET------------ENLRK--ASRYPFVLD-T-----LRSPSVGC  343
hsATL1    KFLEKRL------------KVSGN----QHEELQN-VRKHIHSCFTNISC  266
nsATL1    KFLEKRL------------KVSGN----QHEELQN-VRKHIHSCFTNISC  266
xtATL1    KFLEKRL------------KVSEN----QHEELQN-VRKHIHSCFTNISC  264
drATL1    KILKRRL------------EVSDK----QHPELQS-LRRHISSCFTEVAC  241
hsGBP1    EYLTYSL------------KLKKG--TSQKDETFNLPRLCIRKFFPKKKC  235
hsMFN1    EYMEDVRRQHMERCLHFLVEELKVVNA---LEAQN------RIFFVSAKE  287
hsMFN2    EYMEEVRRQHMERCTSFLVDELGVVDR---SQAGD------RIFFVSAKE  308
caSey1p   ------A------------ELE----HLQFADFFD-----------VTF  249
scFzo1    ---------DKQRCKELILKQIRDLSPETYKRAAD------FVHFVSKNG  411

β8G  α7G
xlRNF112  FLLPHPGKGITG------E--SEGRL-QDMDEDFQESLRS-------YV-  409
hsRNF112  CLLPAPGRRRMN------Q--GHASP-GDTDDDFRHLLGA-------YV-  389
mmRNF112  YLLPAPERQWVN------K--DQASPRGNTEDDFSHHFRA-------YI-  412
nsRNF112  YLMPFPGKALVM------G--TEGTI-AGMDEDFRGFLKD-------YG-  382
amRNF112  YLMPHPGNKFTN------S--SNGTL-ADMDEVFRDSLRD-------YV-  262
gsRNF112  CLLPSRVED----------------T-QDIAEDLGKMYSS-------L--  346
xtRNF112  CLLPHPGKRLLG------D--SQGSL-SDMDEDFKNHLGN-------YI-  376
hsATL1    FLLPHPGLKVAT------NPNFDGKL-KEIDDEFIKNLKI-------LI-  301
nsATL1    FLLPHPGLKVAT------NPNFDGKL-REIDDDFIKNLKI-------LI-  301
xtATL1    FLMPHPGLTVAT------NPNFDGKL-KEIDEEFIKNLKV-------FI-  299
drATL1    FLMPHPGLNVAT------NPKFDGRL-QDITPEFKSSLRS-------LV-  276
hsGBP1    FVFDRPVHRRKL------AQLEKL-QDEELDPEFVQQVAD-------FC-  270
hsMFN1    VLSAR-----------KQKAQGMPESGVALAEGFHARLQEFQNFEQIFEE  326
hsMFN2    VLNAR-----------IQKAQGMPEGGGALAEGFQVRMFEFQNFERRFEE  347
caSey1p   HALNHK----VL------QPKEFGEGINRLDDRLVVSNELFK---PEYH-  285
scFzo1    DELPHYHNENDNEDHGDRKPDDDPYSSSDPDPDEDSLEDSLRNFVL----  457

α7G
xlRNF112  ------------------------SKVVKGICTHIKTN------------  423
hsRNF112  ------------------------SDVLSAAPQHAKSRCQGYWNEGR---  412
mmRNF112  ------------------------LDVLSTAPQHAKSRCQGYWSEGR---  435
nsRNF112  ------------------------NHIARSAGMHVQRN------------  396
amRNF112  ------------------------AGLASEAGTCVRLD------------  276
gsRNF112  --------------------------NSALKYTKED-------------  356
xtRNF112  ------------------------TDLVRGIWRHVKTD------------  390
hsATL1    ------------------------PWLLSPESLDIKE-------------  314
nsATL1    ------------------------PWLLSPKSLDVKE-------------  314
xtATL1    ------------------------PWLLSPENLDVKE-------------  312
drATL1    ------------------------PMLLAPDNLVYKE-------------  289
hsGBP1    ------------------------SYIFSNSKTKTLS-------------  283
hsMFN1    CISQSAVKTKFEQHTIRAKQILATVKNIMDSVNLAAEDKRHYSVEERDQ  376
hsMFN2    CISQSAVKTKFEQHTVRAKQIAEAVRLIMDSLHMAAREQQVYCEEMREER  397
caSey1p   ------------------------HDVPIDGWTMYAERCWEQIETNK---  308
scFzo1    -------KKRSLSKLLPAKTYL--SKLLSDIIMISKSNMKMYSEEEIK--  496

α8G
xlRNF112  -----------------IDGELLTSAH-VFSMLQEF-------------  441
hsRNF112  ---------AVAR----GDRRLLTGQQ-LAQEIKNL-------------  434
mmRNF112  ---------AVAR----GDRRLLTGQQ-LAQEIKNL-------------  457
nsRNF112  -----------------QKNKPLTGTE-LANKIKAL-------------  414
amRNF112  -----------------QKGQALTGKQ-LASKIRDI-------------  294
gsRNF112  -----------------GDQNTLTLEQ-LAKRIKIF-------------  374
xtRNF112  -----------------IHGEKVTCAQ-LEGKLKEF-------------  408
hsATL1    -----------------INGNKITCRG-LVEYFKAY-------------  332
nsATL1    -----------------INGNKITCRG-LVEYFKAY-------------  332
xtATL1    -----------------ISGNRITCRG-LVEYFKAY-------------  330
drATL1    -----------------ISGQRVRARD-LIQYFQSY-------------  307
hsGBP1    -----------------GGIQVNGPR-LESLVLTY-------------  300
hsMFN1    IDRLDFIRNQMNLLTLD---VKKKIKEVTEEVANKVSC--AMTDEICRLS  421
hsMFN2    QDRLKFIDKQLELLAQD---YKLRIKQITEEVERQVST--AMAEEIRRLS  442
caSey1p   ---------DLDLPTQQILVAQFKCDEIVESVFQEF-------------  335
scFzo1    ------INEQLETLRPEILSARAKCNDLTTSVDQMAEQTITMTYNN-TKE  539

α8G  Hinge2  α1M
xlRNF112  --TEVLNLQI---YGFSSEMEMFYAIKNQ------KLMGEIENEFQDFLK  480
hsRNF112  --SGWMGRTG---PGFTSPDEMAAQLHDL------RKVEAAKREEEYVR  473
mmRNF112  --SGWMGKTG---PSFNSPDEMAAQLHDL------RKVEAAKKEEEYVR  496
nsRNF112  --SELMKQYH---TGFSSPIEMAYTFNQN------N-NKMFQEKEKKFIQ  452
amRNF112  --SKYLRDKN---YHFSSLTKMTEAFAQIREDLNKTINKIIMEVENCIL  339
gsRNF112  --TEILK-TS---YGFSSPLEMGITIHNY------KVREIFRRNVRHFLK  412
xtRNF112  --VGLLQKEQ---YSFASPLEMFFTFENR------KMSSVKREGLNHLD  447
hsATL1    --IKIYQG-----EELPHPKSMLQATAEAN---NLAAVATAKDTYNKKME  372
nsATL1    --IKIYQG-----EELPHPKSMLQATAEAN---NLAAVATAKDTYNKRME  372
xtATL1    --IKIYQG-----EELPHPKSMLQATAEAN---NLAAVATAKDLYNKNME  370
drATL1    --MNIYKG-----NELPEPKSMLVATAEAN---HLTAVAAAKELYGQLME  347
hsGBP1    --VNAISS-----GDLPCMENAVLALAQIE---NSAAVQKAIAHYEQQMG  340
hsMFN1    VLVDEFCSEF---HPNPDV---LKIYKS--------------ELNKHIE  450
hsMFN2    VLVDDYQMDF---HPSPVV---LKVYKN--------------ELHRHIE  471
caSey1p   --LAKYQHHFKEVDAAPDFEELGALFADLRQDAF-EDYDASASRYNKAVY  382
scFzo1    ALLNALDVPL---HEYPKYQGLGQIYDFI----F-----STEAFIANQID  577

α2M  Variant linker
xlRNF112  NQSSLTLP----------P---------TYRVKVS---QKFSELLEKFMQ  508
hsRNF112  QQDVATKR----------IFSALRVLPDTVRNLLS---TQKDAILARHGV  510
mmRNF112  QQDIATKR----------IFSALRVLPDTVRNLLS---TQKDAILARHGV  533
nsRNF112  KQDKFSCS----------ILASLRVSPNSYTKRFE---EKQKKLLQQFRD  489
amRNF112  EQDCTQI-----------EAQCLQVPPSKVQQRLE---EKQQQLLQDCAA  376
gsRNF112  EQDLQYCS----------FLKIVQLRSDALTDELY---RQLLQLLEESAR  449
xtRNF112  NLAPASSS----------PLKILGVTPSKVKGRTS---EAAAHFLAVYEE  484
hsATL1    EICGGDKP----------FL-----APNDLQTKHL---QLKEESVKLFRG  404
nsATL1    EVCGGDKP----------FL-----APSDLQNKHL---ELKEEAVRQFRS  404
xtATL1    EVCGGDRP----------FL-----APTDLQQRHQ---HLKEEAVKLFRG  402
drATL1    EVCGGTRP----------YL-----STAHLQTEHL---RVKDKALFQFAA  379
hsGBP1    QK-------------------VQLPTETLQELLDLHRDSEREAIEVFIR  370
hsMFN1    DGMGRNLADRCTDEVNALVLQTQQEIIENLKPLLPAGIQDKLHTLIP---  497
hsMFN2    EGLGRNMSDRCSTAITNSLQTMQQDMIDGLKPLLPVSVRSQIDMLVP---  518
caSey1p   EQKRKKLR----------W-----LINDKLKE------------------  399
scFzo1    ESIGSS-----------------------------ELFA---KQKTDLL-V---  595

α3M  End of crystallized xlRNF112 constructs
xlRNF112  -FVQGSNTSSHDAMLKDLEVRLLEIQEKFCNDETTRFTMNAV-GVGI---  553
hsRNF112  -ALLCKGR---DQTLEALEAELQATAKAFMDSYTMRFCGHLA-AVGG---  552
mmRNF112  -ALLCKER---EQTLEALEAELQAEAKAFMDSYTMRFCGHLA-AVGG---  575
nsRNF112  -SFHGDEPHQTE-FLTCLESYFKYEANNFLEDYRRHFKSSAI-KAGV---  533
amRNF112  -ELRGEERQKQP-FLQELEQRLMEKEKAFVDKYKKKYKNAVS-SANR---  420
gsRNF112  -HLQGDENQKQHLLQE-MLNEAEEEIGGVTSGYTRKRRRTLL-ASA----  492
xtRNF112  -SIKGDDSQGRQLLMEEMESILREAEDKFNDEYSKRFKKCAI-GIGC---  529
hsATL1    VKKMGGEEFS-RRYLQQLESEIDELY----IQYIKHNDSKNIFHAARTPA  449
nsATL1    VKKMGGEEFS-RRYLQQLEAEIDELY----VQYIKHNDSKNIFHAARTPA  449
xtATL1    VKKMGGEEFS-RRYLQQLENEIDELY----VQYIKHNDSKNIFHAARTPA  447
drATL1    KRKMGGEEFT-EKFRKQLEDDLEEVF----TNYQAHNESKNIFKAARTPA  424
hsGBP1    ----SSFKDVDHLFQKELAAQLEKKRDDFCKQNQEASSDRCS-----  408
hsMFN1    --------------CKKFDLSYNLNYHKLCSDFQEDIVFRFS----LGWS  529
hsMFN2    --------------RQCFSLNYDLNCDKLCADFQEDIEFHFS----LGWT  550
caSey1p   --------------------VFDVHAKNLCNTLLEKFEKDLVALKGKDFA  429
scFzo1    -----------------KKIYEIGKNELGDDEMCERVFRSE----LMFR  623

xlRNF112  -------------TAGFGVCGVLGATAARG-------------------  570
hsRNF112  --------------AVGAGLMGLAGGVVGAGM--------------A---  571
mmRNF112  --------------AVGAGLMGLAGGVVGAGM--------------A---  594
nsRNF112  --------------AIGVGAVGLAGGAVGAGI--------------A---  552
amRNF112  --------------KITKKAVNQYEQFMQKQP--------------P---  439
gsRNF112  -------------------------AAILGVAV-------------A---  501
xtRNF112  --------------AIGGGVLSLAGGVVGAAV--------------A---  548
hsATL1    TLFVVIFI---T--YVIAGVTGFIGLDIIASL--------------C---  477
nsATL1    TLFVVIFI---T--YVIAGVTGFIGLDIIASL--------------C---  477
xtATL1    TLFVVIFI---T--YVLAAVTGFIGLDIIASL--------------C---  475
drATL1    VYFACAVI---M--YILSGIFGLVGLYTFANF--------------C---  452
hsGBP1    ALLQVIFSPLEE--EVKAGIYSKPGGY-------------------------  433
hsMFN1    SLVHRFLGP---RNAQR-VLLGLSEPIFQLPRSLASTPTAPTTPATP-DN  574
hsMFN2    MLVNRFLGP---KNSRR-ALMGYNDQVQ---RPIPLTPANPSMPPLPQGS  593
caSey1p   VNVKTLSTKLVEDVNFQVSLMSLQGDLSLDEIILALT--KDIDAIVAKQQ  477
scFzo1    KRKH-LIGKI---RLKVSLSITDLFAPTWKGFLS-YLSWQKPVTAPLPDIE  668

xlRNF112  ---------------------------------TAMLA-AQP---------  578
hsRNF112  ---------------------------------AAALA-AEA---------  579
mmRNF112  ---------------------------------AAALA-AEA---------  602
nsRNF112  ---------------------------------AALLA-GEA---------  560
amRNF112  ---------------------------------AQSLM-V-----------  445
gsRNF112  ---------------------------------APALS-LTA---------  509
xtRNF112  ---------------------------------GTVLA-AEA---------  556
hsATL1    ---------------------------------NM-IM-G-----------  482
nsATL1    ---------------------------------NM-IM-G-----------  482
xtATL1    ---------------------------------NM-IM-G-----------  480
drATL1    ---------------------------------NL-VM-G-----------  457
hsGBP1                                                         -
hsMFN1    A----SQEELMIT---------------------LV--TGLASVTSRTSM  597
hsMFN2    L----TQEEFMVS---------------------MV--TGLASLTSRTSM  616
caSey1p   ------VIELNSI-------------------VNKSVK-KLSASLSKSIQF  502
scFzo1    GQTNEGQIGLMKYLGLKNYPLTQYWSRPSLLFTSKIPTLTLYLFLGSTKVV  718

xlRNF112  -VVAVGERAVAM----EIASGAVSM--LKTGMTAMLG------------  608
hsRNF112  -GMVAAGA----------AVGATGAAVVGGGVGAGLAAT-----------  607
mmRNF112  -GMVAAGA----------AVGATGAAVVGGGVGAGLAAT-----------  630
nsRNF112  -IVLGTGATFAI----GTVAGAGTFGLVGGGVGAGVGS--R---------  594
amRNF112  -------TPYDMQ--QGLEKKRQKL--LHDCRGKLCGDKAS---------  475
gsRNF112  -------LPFPV---AAMVGGMASL-FAGSS----LGA--I---------  533
xtRNF112  -VALVGSTTAAVI--TGAVGGTVTLGAIGTGVGAGVGA--G---------  592
hsATL1    -LTLITLCTWAYI--R-YSGEYRELGAVIDQVAAALWDQ-----------  517
nsATL1    -LTLITLCTWAYI--R-YSGEYRELGAVIDQVAAALWDQ-----------  517
xtATL1    -LTLITLCTWAYI--R-YSGEYRELGAVIDQVAAALWDQ-----------  515
drATL1    -VALLTLALWAYI--R-YSGELSDFGGKLDDFATLLWEK-----------  492
hsGBP1    -------------------------RLFVQKLQDLKKKYYEEPR---------  452
hsMFN1    GIIIVGGVIWKTIGWKL---LSVSL----TMYGALYLYERL---------  631
hsMFN2    GILVVGGVVWKAVGWRL---IALSF----GLYGLLYVYERL---------  650
caSey1p   ELGDPNEETWDNVL-QQFKGVYEKF---GGDFGLGTSSTQNQQAIEKFKF  548
scFzo1    GNIILNGIKL--SSWSSLKKLSVPVIVVGSLLGLTYLIHLDL---------  757
```

```
xlRNF112   ---RFF--------------------------------------------   611
hsRNF112   -VGCME--------------------------------------------   612
mmRNF112   -VGCME--------------------------------------------   635
nsRNF112   -IGSSK--------------------------------------------   599
amRNF112   -QRSELENLTQKLLRKEEVFMDAYRN-----------VY-----------   502
gsRNF112   -IRSQI--------------------------------------------   538
xtRNF112   -VGGAI--------------------------------------------   597
hsATL1     ----------------------GSTN-----------EA-----------   523
nsATL1     ----------------------GNTN-----------EV-----------   523
xtATL1     ----------------------VSS------------IA-----------   520
drATL1     ----------------------FMRP-----------IY-----------   498
hsGBP1     -KGIQAEEILQTYLKSKESMTDAILQTDQTLTEKEKEIEVERVKAESAQA   501
hsMFN1     -SWTTHAK------------------------------------------   638
hsMFN2     -TWTTKAK------------------------------------------   657
caSey1p    KSWCQFYDVTHKLISREKLL------------------------------   568
scFzo1     -PRALPMNL-----------------------------------------   765
```

```
xlRNF112   ---------R----------------------------------------   612
hsRNF112   ---------------KEEDER-----------LLEGDREPLLQEE-----   631
mmRNF112   ---------------KEEDER-----------VQGGDREPLLQEE-----   654
nsRNF112   ---------SNNNKNEAKDDN-----------EIEDEEEENVSDEK----   625
amRNF112   KGTV----FSVNGKIIEKAKR-----------EYEH----FLQEQL----   529
gsRNF112   ---------TDRKKN-----------------------------------   544
xtRNF112   ---------ANRKTEAEQQTT-------------EEGGDGSGTDSQ----   621
hsATL1     LYKL----YSAAATHRHLY-------------------------------   538
nsATL1     SQLI----ITQKQFLLHQYTY-----------------------------   540
xtATL1     LYKL----YSAAATHRHLY-------------------------------   535
drATL1     HGCM------EKGI-HHVATH-----------------------------   512
hsGBP1     SAKMLQEMQRKNEQMMEQKERSYQEHLKQLTEKMENDRVQLLKEQE----   547
hsMFN1     ---------------ERAFKQQFVNY--------ATEKLRMIV-------   658
hsMFN2     ---------------ERAFKRQFVEH--------ASEKLQLVI-------   677
caSey1p    --ALLQDRFD--------DKF-----------RYDENGLPKLYLNEQDLE   597
scFzo1     ---------------SIKYKRKLQEL--------DY-----I--------   779
```

```
xlRNF112   --------------------------------------------------   -
hsRNF112   --------------------------------------------------   -
mmRNF112   --------------------------------------------------   -
nsRNF112   -------------------------SLIS---------------------   629
amRNF112   -------------------------QDITPKFSIFLWSVKKNQELGKKKH   554
gsRNF112   --------------------------------------------------   -
xtRNF112   -------------------------QLVAPKK------------------   628
hsATL1     ---------------------------------------HQAFPTPKS--   547
nsATL1     ---------------------------------------MNDYACGSI--   549
xtATL1     ---------------------------------------HHAFPVPQA--   544
drATL1     ---------------------------------------ATEMAVGGG--   521
hsGBP1     -------------------------RTLALKLQ-----------------   555
hsMFN1     --------------------------------------------------   -
hsMFN2     --------------------------------------------------   -
caSey1p    KTFAVAKQHALQVLPILTFAKLADGSEIVPDYDIFDSKLREQFLGGYDDS   647
scFzo1     --------------------------------------------------   -
```

```
xlRNF112   --------------------------------------------------   -
hsRNF112   --------------------------------------------------   -
mmRNF112   --------------------------------------------------   -
nsRNF112   --------------------------------------------------   -
amRNF112   -QELLHSYLKQLQGEEREKNASTEKLKQKLAEEERNFRRKCNELLQ---N   600
gsRNF112   --------------------------------------------------   -
xtRNF112   --------------------------------------------------   -
hsATL1     ----EST-------EQS----EKKKM------------------------   558
nsATL1     ----LLQ-------GIC----ITGSFN-----------------------   561
xtATL1     ----DAA-------EET----ERKHI------------------------   555
drATL1     ----AASYR-----SQTSVNASNGKVKRS---------------------   541
hsGBP1     -------------E-------QEQLLKEGFQKESRIMKNEIQDLQT---K   582
hsMFN1     ----------SSTSANC-----SHQVKQQI---ATTFARLCQQ----VDI   686
hsMFN2     ----------SYTGSNC-----SHQVQQEL---SGTFAHLCQQ----VDV   705
caSey1p    DDEEDHCFAEIITEQEK------SEVL-------AKFKKEVDAKYI---E   681
scFzo1     ----------HLNAQRT-----SNEVRDVLRVPTREILRSCEIIMDKKQI   814
```

```
xlRNF112   --------------------------------------------------   -
hsRNF112   --------------------------------------------------   -
mmRNF112   --------------------------------------------------   -
nsRNF112   --------------------------------------------------   -
amRNF112   AKDT------RTTVVLGTVGVVVAGGAIVAFILSPALMFK-------AAV   637
gsRNF112   --------------------------------------------------   -
xtRNF112   --------------------------------------------------   -
hsATL1     --------------------------------------------------   -
nsATL1     --------------------------------------------------   -
xtATL1     --------------------------------------------------   -
drATL1     --------------------------------------------------   -
hsGBP1     MRR-------RKACTIS---------------------------------   592
hsMFN1     TQKQLEEEIARLPKEIDQLEKIQN---NSKLLRNKAVQLENELENFTKQF   733
hsMFN2     TRENLEQEIAAMNKKIEVLDSLQS---KAKLLRNKAGWLDSELNMFTHQY   752
caSey1p    TKRSIVQHITQIPYYIYLIILVLGWNEFMAIIRNP-LFFSLSIV-LGATV   729
scFzo1     TKKELENK--------------------------------KESNLLSIKF   832
```

```
xlRNF112   --------------------------------------------------   -
hsRNF112   --------------------------------------------------   -
mmRNF112   --------------------------------------------------   -
nsRNF112   --------------------------------------------------   -
amRNF112   SVL-----------------------------------------------   640
gsRNF112   --------------------------------------------------   -
xtRNF112   --------------------------------------------------   -
hsATL1     --------------------------------------------------   -
nsATL1     --------------------------------------------------   -
xtATL1     --------------------------------------------------   -
drATL1     --------------------------------------------------   -
hsGBP1     --------------------------------------------------   -
hsMFN1     LPSSNEES------------------------------------------   741
hsMFN2     LQPSR---------------------------------------------   757
caSey1p    YVLYYLGLLRPALVVAQRTMDEVIVMAKTKLREVLIDDHEVTGRQLNKMA   779
scFzo1     FQSLYEGTVAQKLMVEEINLD-----------------------------   853
```

```
xlRNF112   -----------   -
hsRNF112   -----------   -
mmRNF112   -----------   -
nsRNF112   -----------   -
amRNF112   -----------   -
gsRNF112   -----------   -
xtRNF112   -----------   -
hsATL1     -----------   -
nsATL1     -----------   -
xtATL1     -----------   -
drATL1     -----------   -
hsGBP1     -----------   -
hsMFN1     -----------   -
hsMFN2     -----------   -
caSey1p    GSKENIELDDM   790
scFzo1     -------ID--   855
```

▼ Arg-finger
▲ Key resiudes involved in dimerization
● Key resiudes involved in conformational change
◆ Hydrophobic core of MD
★ Ubiquitination sites

Fig. S3. Sequence alignment of RNF112 and dynamin superfamily members.

Amino acid sequences of *Xenopus laevis* (xl) RNF112 (UniProt accession Q2VPQ0), *Homo sapiens* (hs) RNF112 (Q9ULX5), *Mus musculus* (mm) RNF112 (Q96DY5), *Notechis scutatus* (ns) RNF112 (A0A6J1VXM5), *Alligator mississippiensis* (am) RNF112 (A0A151MVZ5), *Geotrypetes seraphini* (gs) RNF112 (A0A6P8RFL9), *Xenopus tropicalis* (xt) RNF112 (F7DSK6), hsATL1 (Q8WXF7), nsATL1 (A0A6J1UFK8), xtATL1 (F7DVU9), *Drosophila melanogaster* (dr) ATL1 (Q9VC57), hsGBP1 (P32455), hsMFN1 (Q8IWA4), hsMFN2 (O95140), *Saccharomyces cerevisiae* (sc) Fzo1 (P38297) and *Candida albicans* (ca) Sey1p (Q9C0L9) aligned using Clustal W. Residues with a conservation of 100% are in red shades, > 80% in green shades, and 50% in grey shades. α-Helices are shown as cylinders and β-strands as arrows for xlRNF112 and specified and colored as in **Fig. 1*B***. The G1–G4 elements and key residues are indicated.

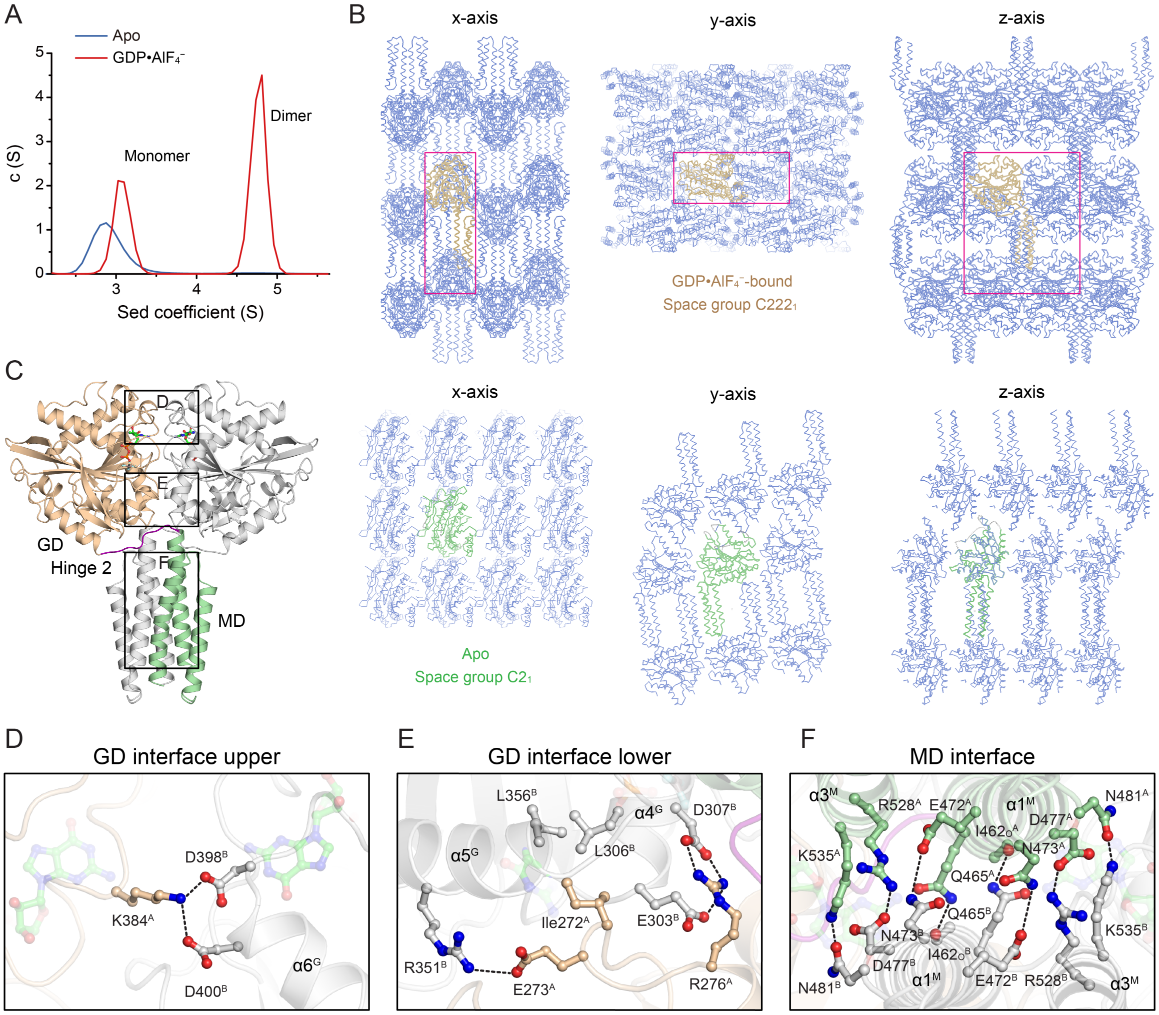

Fig. S4. Dimerization of RNF112$_T$ in the transition-like state.

(A) AUC results for RNF112$_T$ in the absence or presence of GDP•AlF_4^-.

(B) Different crystal packing of RNF112$_T$ in the apo and GDP•AlF_4^--bound forms.

(C–F) Extra intermolecular interactions of the dimeric interface of RNF112$_T$ in the transition state (other than those shown in **Fig. 3 *B*–*D***), including those in the GD **(D** and **E)** and MD **(F)**.

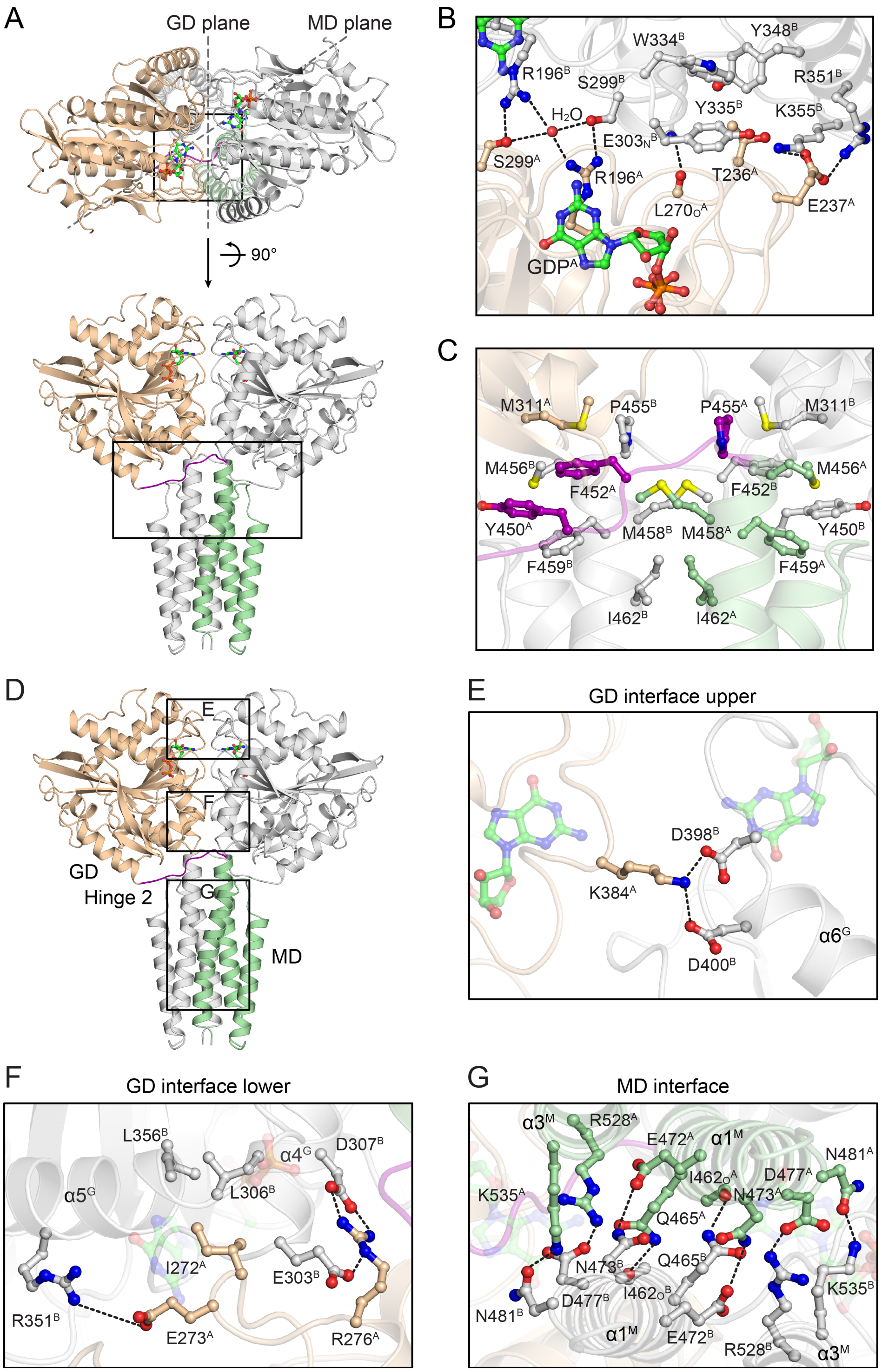

Fig. S5. Dimerization of $RNF112_T$ in GDP-bound form 1 state.

(A–C) The dimeric interface of GDP-bound form 1 $RNF112_T$ around the nucleotide binding site and the crossover Hinge 2 loops. Corresponding to **Fig. 3 *B*–*D*** showing the dimeric interface of $RNF112_T$ in the transition state.

(D–G) Extra intermolecular interactions of the dimeric interface of $RNF112_T$ in GDP-bound form 1. Corresponding to **Fig. S4 *C*–*F***.

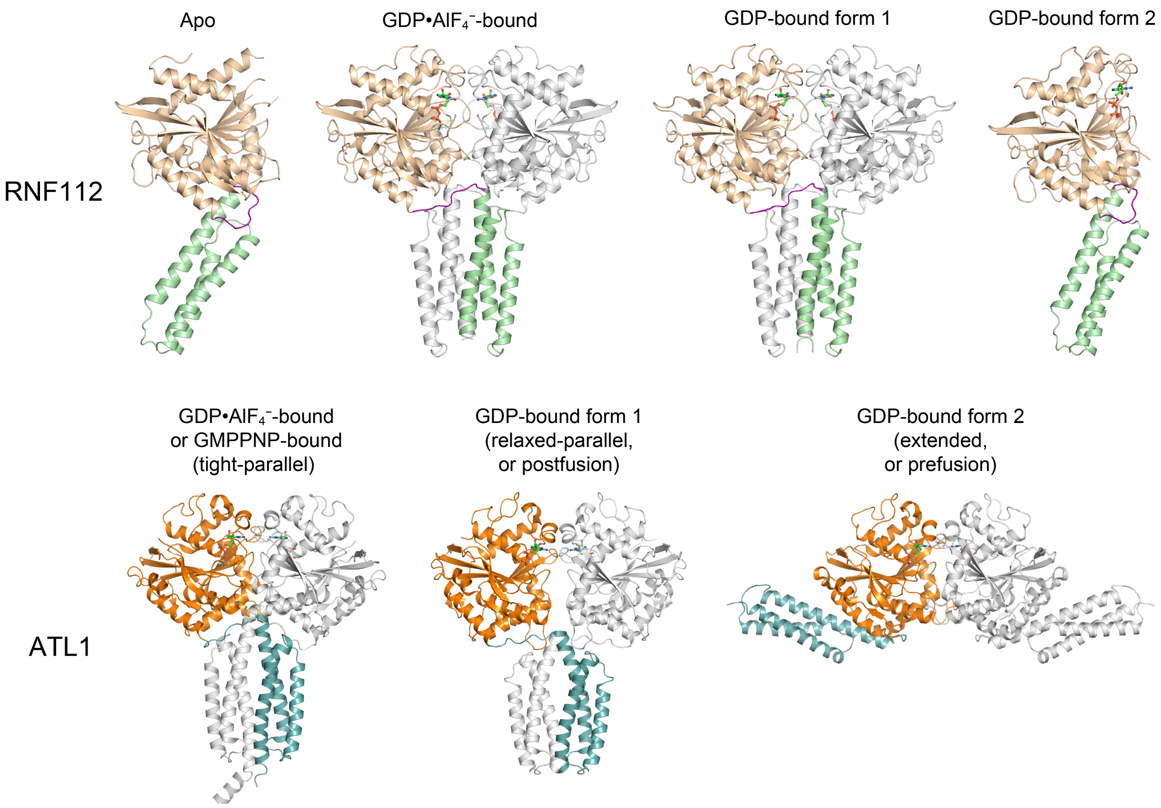

Fig. S6. Conformational changes in RNF112 and ATL1 in different nucleotide binding states.

Structures of RNF112$_T$ in the apo, GDP•AlF4⁻-bound, GDP-bound form 1, and GDP-bound form 2 states are colored as in **Fig. 3*B***. The domains of RNF112T are colored as in **Fig. 1*A***. For ATL1, structures of GDP•AlF4⁻-bound ATL1 (PDB code 4IDO) or GMPPNP-bound ATL1 (4IDN), GDP-bound form 1 ATL1 (3Q5D), and GDP-bound form 2 ATL1 (3Q5E) are shown. The GD and MD of one ATL1 protomer are colored orange and light teal, respectively, and the other protomer is colored gray. Nucleotides are shown as ball-and-stick models.

A

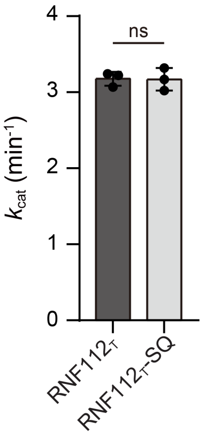

B

	Labeling efficiency	
	LD555	LD655
ATL1$_{cyto}$-TK	78.6%	70.7%
RNF112$_{T}$-SQ	73.6%	70.1%

Fig. S7. Quality control of RNF112$_{T}$ construct used in the FRET assay

(A) The GTP turnover rate of RNF112$_{T}$-SQ was similar to that of WT RNF112$_{T}$. Data are presented as mean ± SD (n = 3). ns, not significant.

(B) Labelling efficiency was calculated as [Fluorophore]/[protein]. [Fluorophore] and [protein] denote the molar concentrations of fluorophore (LD555 or LD655) and protein, respectively.

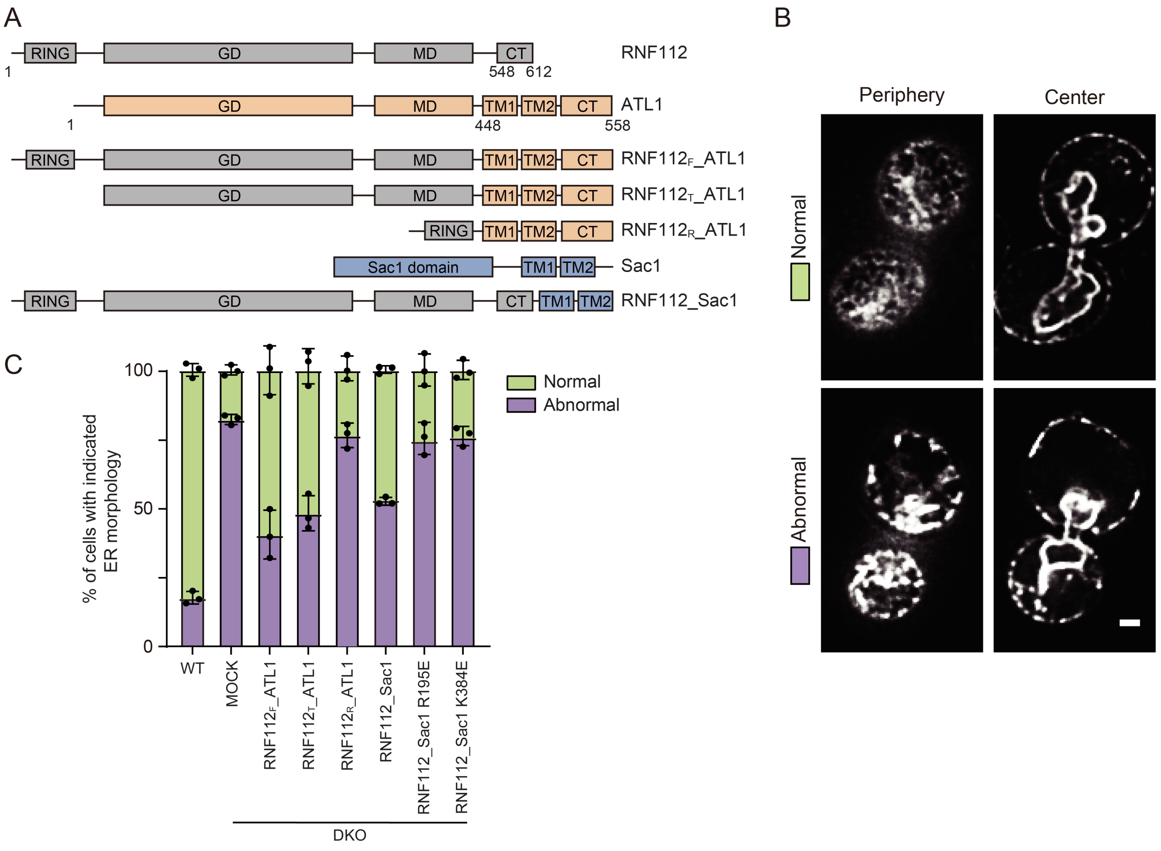

Fig. S8. Functional analysis of RNF112$_T$_ATL1 chimera in yeast cells.

(A) Schematic representation of the organization of RNF112$_T$_ATL1 and RNF112_Sac1. TM, transmembrane domain. The borders of each element are indicated by residue numbers.

(B) Representative fluorescence image showing the fusogenic potential of engineered RNF112 for the ER. A GFP-fusion protein containing the ER protein Sec63p was expressed in yeast cells lacking Sey1p and Yop1p (*sey1Δyop1Δ* cells) to visualize the ER morphology. The microscope focused on either the center or the periphery of the cell. ER morphology was categorized into two classes. Scale bar = 1 µm.

(C) Quantification of engineered RNF112-mediated ER fusion. The ER morphology was analyzed at the periphery of the WT yeast cells or the *sey1Δyop1Δ* yeast cells (DKO) expressing the empty vector or the indicated chimera under the control of the GAL promoter. Data are presented as mean ± SD (n = 469, 474, 352, 374, 395, 344, 350, and 359 cells from left to right).

A

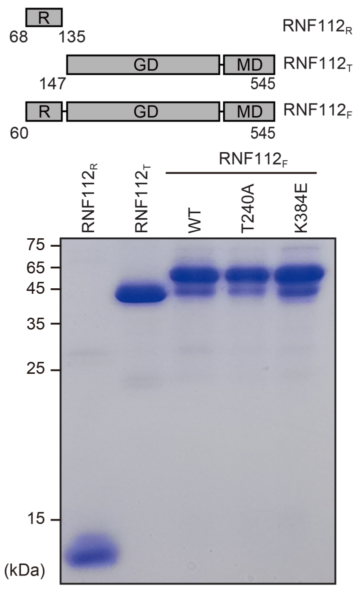

B

Amino acid	GlyGly(K) Probabilities
K136	K(1)SDAMOENLTAAEPDHPIOLVWTDVNGR
K228/K241	SMEMDEEISLGADDEPLK(1)GFK(0.99)WSPGTETTTK(0.011)
K384	CFLLPHPGK(1)GITGESEGR
K466	NOK(1)LMGEIENEFODFLK
K480	LMGEIENEFODFLK(1)NOSSLTLPPTMR
K523	FMQFVQGSNTSSHDAMLK(1)DLEVR
K535	LLEIOEK(1)FCNDFTTR

C

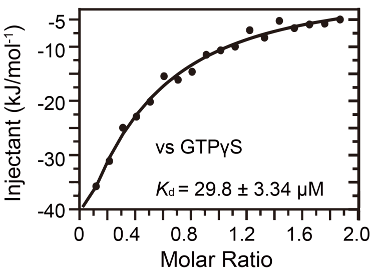

D

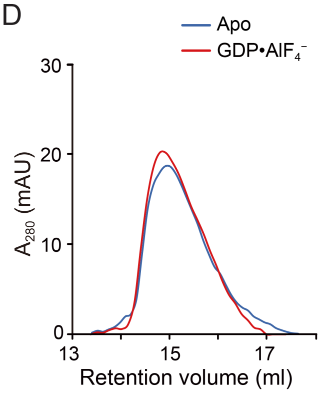

E

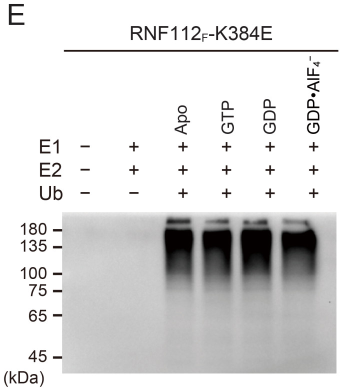

Fig. S9. Biochemical properties of RNF112F mutants.

(A) Coomassie blue staining of purified RNF112 constructs after SDS-PAGE.

(B) Auto-ubiquitination sites of RNF112F identified by mass spectrometry.

(C) Binding affinity of RNF112F(T240A) for GTPγS as measured by isothermal titration calorimetry (ITC).

(D) Dimerization properties of RNF112F(T240A) in the apo and GDP•AlF4$^-$-bound states as assayed by analytical gel filtration. mAU, milli-absorption units.

(E) Auto-ubiquitination of RNF112F(K384E) in the absence and presence of different guanine nucleotides.

Table S1. Crystallographic data collection and refinement statistics.

State	$RNF112_T$ Apo	$RNF112_T$ GDP•AlF_4^-	$RNF112_T$ GDP-bound form1	$RNF112_T$ GDP-bound form2
PDB code	9JJU	9JJV	9JJW	9JJX
**Data collection**				
Data set	Native	Native	Native	Native
Space group	C2	$C222_1$	$C222_1$	P1
Cell dimensions				
a, b, c (Å)	243.5, 39.8, 48.8	91.6, 148.1, 71.3	91.5, 147.4, 71.1	39.4, 48.4, 124.0
α, β, γ (°)	90, 100.04, 90.00	90, 90, 90	90, 90, 90	96.4, 96.3, 90.4
Wavelength (Å)	0.97918	0.97918	0.97853	0.97918
Resolution (Å)	119.88–2.09 (2.20–2.09)*	77.88–1.88 (1.93–1.88)	77.71–2.09 (2.20–2.09)	122.49–2.73 (2.88–2.73)
R_{sym}	0.059 (0.512)	0.092 (1.013)	0.068 (0.664)	0.124 (0.450)
$I/\sigma(I)$	8.3 (2.5)	14.5 (2.0)	16.5 (2.6)	5.7 (2.6)
CC1/2	0.998 (0.922)	0.999 (0.609)	0.999 (0.819)	0.977 (0.860)
Completeness (%)	95.6 (98.4)	99.2 (92.8)	99.9 (99.9)	98 (98.2)
Redundancy	3.8 (3.3)	11.5 (6.1)	5.8 (5.4)	3.2 (3.2)
**Refinement**				
No. reflections	26249 (2650)	39402 (3638)	28814 (2850)	23414 (2385)
Resolution (Å)	27.22–2.09 (2.17–2.09)	35.66–1.88 (1.95–1.88)	43.28–2.09 (2.17–2.09)	31.45–2.73 (2.83–2.73)
R_{work}/R_{free}	0.227/0.231	0.187/0.224	0.184/0.221	0.244/0.295
No. atoms				
Protein	2,898	3,233	3,231	6,432
Ligand/ion	0	7	7	27
Water	71	293	221	51
B-factors				
Protein	65.19	37.26	45.27	75.33
Ligand/ion	0	24.54	42.45	97.01
Water	60.90	40.74	49.64	66.82
R.m.s. deviations				
Bond lengths (Å)	0.008	0.009	0.008	0.003
Bond angles (°)	0.92	1.08	0.96	0.55
Ramachandran				
Favored (%)	96.78	97.45	97.96	93.32
Outliers (%)	0.29	0.00	0.00	0.26

*Values in parentheses are for highest-resolution shell
